# Human Oocyte-Derived Methylation Differences Persist in the Placenta Revealing Widespread Transient Imprinting

**DOI:** 10.1371/journal.pgen.1006427

**Published:** 2016-11-11

**Authors:** Marta Sanchez-Delgado, Franck Court, Enrique Vidal, Jose Medrano, Ana Monteagudo-Sánchez, Alex Martin-Trujillo, Chiharu Tayama, Isabel Iglesias-Platas, Ivanela Kondova, Ronald Bontrop, Maria Eugenia Poo-Llanillo, Tomas Marques-Bonet, Kazuhiko Nakabayashi, Carlos Simón, David Monk

**Affiliations:** 1 Imprinting and Cancer group, Cancer Epigenetic and Biology Program, Institut d’Investigació Biomedica de Bellvitge, Hospital Duran i Reynals, Barcelona, Spain; 2 Laboratoire GReD, CNRS, UMR6293, Clermont-Ferrand, France; 3 Centre for Genomic Regulation (CRG), The Barcelona Institute of Science and Technology, Dr. Aiguader 88, Barcelona, Spain; Universitat Pompeu Fabra (UPF), Barcelona, Spain; 4 Fundación IVI-Instituto Universitario IVI- INCLIVA, Department of Obs/Gyn, Valenica University, Valencia, Spain; 5 Department of Maternal-Fetal Biology, National Research Institute for Child Health and Development, Tokyo, Japan; 6 Neonatal service, Hospital Sant Joan de Déu, BCNatal Hospital Sant Joan de Déu i Clínic, Universitat de Barcelona, Barcelona, Spain; 7 Biomedical Primate Research Center (BPRC), Rijswijk, The Netherlands; 8 Institute of Evolutionary Biology (UPF-CSIC), PRBB, Barcelona, Spain; 9 Catalan Institute of Research and Advanced Studies, (ICREA), Passeig de Lluís Companys, Barcelona, Spain; 10 Centro Nacional de Analisis Genomico (CRG-CNAG), Barcelona, Spain; University of Pennsylvania, UNITED STATES

## Abstract

Thousands of regions in gametes have opposing methylation profiles that are largely resolved during the post-fertilization epigenetic reprogramming. However some specific sequences associated with imprinted loci survive this demethylation process. Here we present the data describing the fate of germline-derived methylation in humans. With the exception of a few known paternally methylated germline differentially methylated regions (DMRs) associated with known imprinted domains, we demonstrate that sperm-derived methylation is reprogrammed by the blastocyst stage of development. In contrast a large number of oocyte-derived methylation differences survive to the blastocyst stage and uniquely persist as transiently methylated DMRs only in the placenta. Furthermore, we demonstrate that this phenomenon is exclusive to primates, since no placenta-specific maternal methylation was observed in mouse. Utilizing single cell RNA-seq datasets from human preimplantation embryos we show that following embryonic genome activation the maternally methylated transient DMRs can orchestrate imprinted expression. However despite showing widespread imprinted expression of genes in placenta, allele-specific transcriptional profiling revealed that not all placenta-specific DMRs coordinate imprinted expression and that this maternal methylation may be absent in a minority of samples, suggestive of polymorphic imprinted methylation.

## Author Summary

Differences in gamete DNA methylation is subject to genome-wide reprogramming during preimplantation development to establish an embryo with an epigenetic state compatible with totipotency. DNA sequences associated with imprinted differentially methylated regions (DMRs) are largely protected from this process, retaining their parent-of-origin epigenetic marks. By comparing the methylation profiles of human oocytes, sperm, blastocysts and various somatic tissues including placenta, we observe hundreds of CpG island sequences that maintain methylation on their maternal allele in blastocysts and placenta indicative of incomplete reprogramming. In some cases this maternal methylation influence transcription of nearby genes, revealing transient imprinting in embryos after genome-activation and in placenta. Strikingly, these placenta-specific DMRs are polymorphic between placenta samples with a minority of samples being robustly unmethylated on both alleles.

## Introduction

In mammals, DNA methylation of CpG dinucleotides has been shown to play critical roles in many developmental processes including cellular differentiation, X chromosome inactivation and genomic imprinting. DNA methylation patterns are initially established by the *de novo* DNA methyltransferase DNMT3A [1], with the methylation profile faithfully maintained during DNA replication by the maintenance methyltransferase DNMT1-UHRF1 complex [2].

It has recently been shown that the gametes from both mouse and humans possess large intervals of opposing methylation [3–7]. Within a few hours after fertilization, a wave of global epigenetic reprogramming ensures that methylation at the blastocyst stage is at their lowest level, erasing the majority of this gametic epigenetic information [3, 5, 7]. However, some specific sequences survive this demethylation process, specifically those located within imprinted regions and certain repeat subtypes. Imprinted genes are only transcribed from one parental allele leading to parent-of-origin specific expression, with allelic expression directly controlled by allelic methylation [8]. To date all imprinted domains contain at least one differentially methylated region (DMR) that acquires methylation during gametogenesis (germline DMR, or gDMR), and maintained throughout development. Some imprinted loci also contain DMRs that become allelically methylated in the embryonic diploid genome (somatic DMRs, or sDMR) which are under the hierarchical influence of gDMRs [4, 9, 10]. Recently, transiently methylated germline DMRs (tDMRs) have been identified in mice that are indistinguishable from ubiquitous imprinted gDMRs in gametes and preimplantation embryos [11]. The maternally methylated tDMRs described in mouse subsequently gain methylation on their paternal alleles at implantation, having first survived the post-fertilization demethylation process. This reprogramming to a totipotent state starts in the male pronucleus with TET3-mediated conversion of 5-methylcytosine (5mC) to 5-hydroxymethylcytosine (5hmC) [12] with subsequent replication-dependent dilution of methylation of both maternal and paternal genomes occurring during the first 2 days of human development [5, 7, 13]. Unlike mice, in humans it is currently unknown how many germline differences survive embryonic reprogramming and persist in humans, either as ubiquitous imprinted gDMRs or tDMRs. However initial screens suggest that oocyte-derived tDMRs may be present to the blastocyst stage [7, 14].

Here, we present the data describing the fate of germline-derived methylation in humans. Using publically available methyl-seq datasets from gametes, preimplantation embryos, placenta and somatic tissues, we identify 53,549 methylation differences between gametes, the majority being methylated in the sperm and not in oocytes. With the exception of a few paternally methylated gDMRs associated within known imprinted domains, we demonstrate that sperm-derived methylation is reprogrammed by the blastocyst stage. In contrast a large number of oocyte-derived methylation differences survive to the blastocyst stage, persisting as maternally methylated DMRs in the placenta only, expanding the number of placenta-specific DMRs reported using high-density array based screens [10, 15–17]. Furthermore, we demonstrate that this phenomenon is exclusive to humans and non-human primates since no placenta-specific maternal methylation was observed in other mammalian species. Utilizing single cell RNA-seq datasets from human preimplantation embryos [18] we show that following embryonic genome activation the maternally methylated gDMRs orchestrate imprinted expression in preimplantation embryos. However, despite showing imprinted expression of many genes, transcriptional profiling revealed that not all placenta-specific maternally methylated DMRs coordinate imprinted expression suggesting differential reading of this epigenetic mark during human embryonic development.

## Results

### Identification of persisting gDMRs in preimplantation human embryos

Transient maternally inherited monoallelic methylation has been previously observed in mouse. To identify candidate loci in humans we searched for regions that are differentially methylated between sperm and oocytes. Using defined criteria (see methods) we identified 5, 438 oocytes and 48, 111 sperm-derived DMRs.

**Fig 1:**
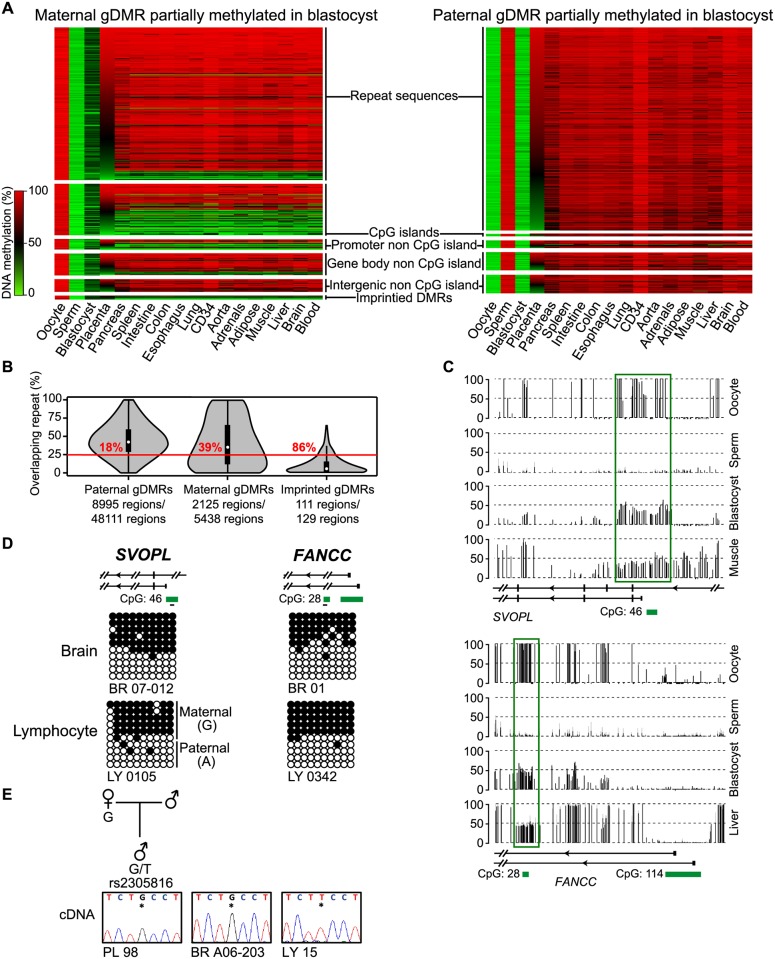
Methylation profiling of human gametes, embryos and tissues. (**A**) Heatmap of all 25 CpG dinucleotide tiles with maternal (left) and paternal (right) germline-derived methylation maintaining an intermediate state in blastocysts and arranged in descending order according to their placenta methylation profile. Tiles were partitioned according to their hypo- (<20%), hyper- (>80%) or intermediate (>20%, <80%) methylation. Genomic features are included as separate heatmaps. (**B**) Violin plots classified as the % repetitive sequences per 20 CpG dinucleotide tile for the paternally methylated, maternally methylated and known imprinted DMRs. The numbers in red represent the tiles with unique sequences (defined as >25% repeats). (**C**) Two novel maternally methylated ubiquitous DMRs associated with the *SVOPL* and *FANCC* genes exhibit promoters that are unmethylated in sperm, hypermethylated in oocytes and intermediately methylated in blastocysts, placenta and somatic tissue in methyl-seq datasets. The vertical black lines in the methyl-seq tracks represent the mean methylation value for individual CpG dinucleotides. Green boxes highlight the position of the gDMRs. (**D**) Bisulphite PCR and subcloning was used for confirmation. Each circle represents a single CpG dinucleotide on a DNA strand. (•) Methylated cytosine, (o) unmethylated cytosine. Each row corresponds to an individual cloned sequence. If heterozygous for a SNP, the parental-origin of methylation is indicated. For clarity only the first 10 CpG dinucleotides are shown. (E) Allelic RT-PCR for *SVOPL* reveals transcription from the maternal allele in placenta and monoallelic expression in brain and leukocytes.

A high proportion of regions methylated in sperm and hypomethylated in oocytes were intergenic or map to repeat elements, consistent with previous observations [5]. In contrast, oocyte-specific DMRs were more uniformly distributed throughout the genome, often overlapping promoter CpG islands. Eighty percent of the oocyte-derived DMRs (n = 4, 352) remain partially methylated at the blastocyst stage, which is consistent with methylation dynamics during the progression of cleavage stage embryos to blastocysts [6, 7] with very few sperm-derived DMRs surviving to the blastocyst stage (1%, n = 517) (Fig 1A and 1B). This reprogramming is particularly evident when the size of the gDMRs surviving to the blastocyst stage is taken into consideration. In total ~7 Mb of the human genome encompasses oocyte-derived gDMRs of which 74% is hemimethylated in preimplantation embyros, whereas ~2.7 Mb is covered by sperm-derived gDMRs of which only 11% is hemimethylated at the same developmental stage. Therefore, maternal gDMRs are lost after the blastocyst stage whereas the methylation at paternal gDMRs is largely removed during preimplantation stages, possible occurring before the first cleavage division arguing against a simple replication-dependent demethylation of the maternal genome during preimplantation development.

### Selected survival of imprinted DMRs

Numerous studies have shown that gDMR that persist uniformly in somatic tissues act as imprinting control regions. To date 49 ubiquitous imprinted DMRs have been identified in humans using high-density methylation arrays [10]. To determine if additional imprinted DMRs are present in the human genome, we determined the methylation profile of the oocyte and sperm-derived gDMRs that are present with preserved methylation in methyl-seq datasets in blastocysts, placenta and 14 different somatic tissues. We observe only one sperm-derived region mapping to a known paternally methylated DMR in > 12 tissues, the *H19* gDMR on chromosome 11. The only additional known paternally methylated DMR originating from sperm in humans, the *IG*-DMR on chromosome 14, was differently methylated between gametes but was partially methylated in blastocysts and five somatic tissues only. Using the same criteria we observe 60 oocyte-derived DMRs in >12 tissues, including 25 known maternally methylated imprinted DMRs (S1 Table). Of the remaining intervals not associated with known imprinted gDMRs, we confirm *FANCC* and *SVOPL* as being novel ubiquitous imprints (S1 Fig; Fig 1C and 1D). Using allele-specific RT–PCR that incorporated a coding SNP within exon 5, we observed maternal expression of *SVOPL* in placenta and monoallelic expression in brain and leukocytes (Fig 1E). Unfortunately we could not identify any informative samples to allow for the allelic expression of *FANCC* to be ascertained.

### Identification of widespread maternally methylated placenta-specific DMRs

**Fig 2:**
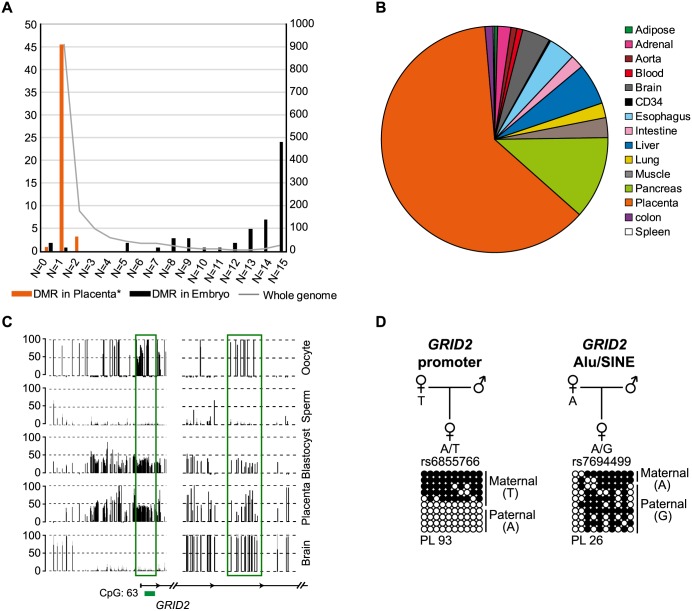
Analysis of tissue-specific maintenance of germline methylation in different tissues. (**A**) A bar graph showing the fate of gDMRs in tissues. The bars represent the profiles of known ubiquitous (black) and placenta-specific (orange) gDMRs with numbers corresponding to the left y-axis. The superimposed line graph represent the profile of all remaining germline difference that are maintained to the blastocyst stage and correspond to the right y-axis. * placenta-specific DMRs identified by Court (2014) [10] and Sanchez-Delgado (2015) [15]. (**B**) A pie graph showing the distribution of individual tissues maintaining a partially methylated profile. (**C**) The *GRID2* gene exhibits high inter- and intragenic methylation and several regions with oocyte-derived methylation. The methyl-seq data reveals that a 1.9 kb region overlapping the promoter remains an imprinted gDMR in placenta while it is demethylated in all other tissues. A second oocyte-derived gDMR, consisting largely of an Alu/SINE repeat, becomes fully methylated in all tissues analysed. The vertical black lines in the methyl-seq tracks represent the mean methylation value for individual CpG dinucleotides. A green box highlights the position of the gDMR. (**D**) Bisulfite PCR and subcloning on heterozygous placenta DNA samples for the *GRID2* promoter and intragenic regions. Each circle represents a single CpG dinucleotide on a DNA strand. (•) Methylated cytosine, (o) unmethylated cytosine. Each row corresponds to an individual cloned sequence. If informative for a SNP, the parental-origin of methylation is indicated. For clarity only the first 10 CpG dinucleotides are shown.

To determine if germline-derived DMRs are maintained in a tissue-specific fashion we screened for loci partially methylated in only one tissue (Fig 2A and 2B). This analysis revealed that 551 of the partially methylated regions in blastocysts inheriting methylation from the oocyte survived only in the placenta, whereas only 38 regions inheriting methylation from sperm were identified in this extra-embryonic tissue (S2 Table). Since standard bisulphite conversion based technologies cannot distinguish between 5mC and 5hmC, we utilized methylation-sensitive genotyping assays that can distinguish these two forms based on the addition of a glucose moiety to yield glucosyl-5-hydroxymethylcytosine. This, combined with allele-specific bisulphite PCR, revealed no novel paternally methylated placenta-specific gDMRs since all candidates were mosaically methylated, but maternal placenta-specific gDMRs were abundant and specifically associated with 5mC (S2, S3 and S4 Figs; S2 and S3 Tables).

The fate of 5mC at maternal placenta-specific gDMRs in somatic tissues was largely influenced by sequence content. The confirmed placenta-specific maternal methylation regions were almost always high CG content intervals robustly unmethylated in somatic tissues, whereas hypermethylated loci in somatic tissues were often false positives being partially methylated regardless of the parental allele, with the exceptions of four loci which we confirm as maternally methylated (*TMEM247*, *GPR1-AS1*, *ZFAT*, and *C19MC*) (S5 and S6 Figs) [19, 20]. This reflects the general methylation status of the placenta, which is relatively hypomethylated across the genome, including repeat elements [21, 22]. For example the *GRID2* gene is associated with two maternally methylated gDMRs with different genomic content. The promoter CpG island is robustly methylated on the maternal allele in placenta and is unmethylated in somatic tissues, whereas an intergenic region within intron 3, consisting of an Alu/SINE repeat, is a gDMR with a mosaic methylation profile in placenta that is fully methylated in all somatic tissues (Fig 2C and 2D).

We observe robust maternal methylation associated with multiple members of two large gene families, the fibroblast growth factors (*FGF8*, *FGF12* and *FGF14*) and calcium channel, voltage-dependent channel subunits (*CACNA1A*, *CACNA1C*, *CACNA1E* and *CACNA1I*) as well as several gene involved in epigenetic regulation (*JMJD1C* and *DNMT1*) and microRNA processing (*LIN28B* and *EIF2C1*) (S3 and S4 Figs). These results therefore reveal that placenta-specific gDMRs are much more abundant in the human genome than previously reported.

### Confirmation of germline profiles and methylation asymmetries in human embryos

**Fig 3:**
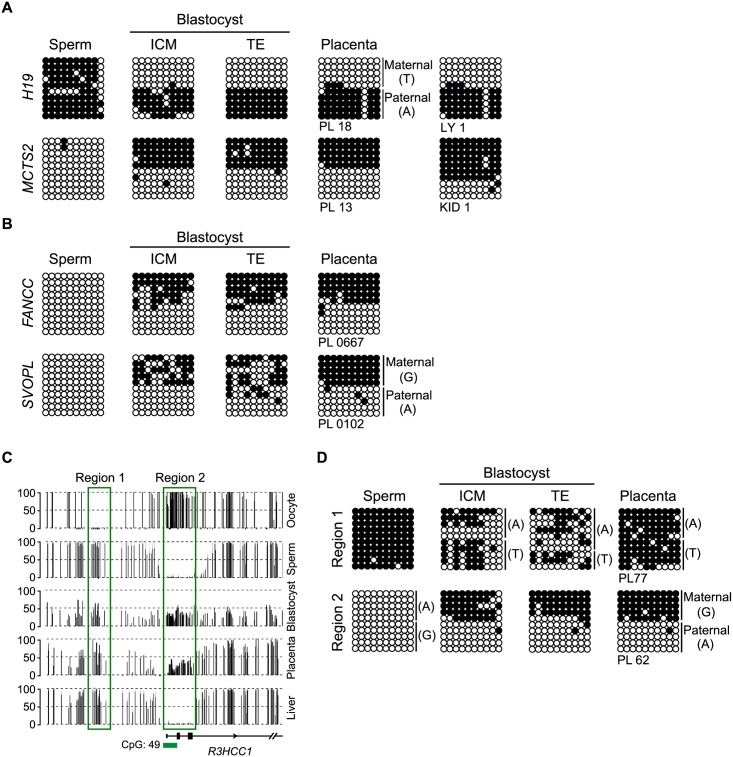
Methylation profiling of opposing gDMRs using bisulphite PCR in human gametes and blastocysts. (**A**) The confirmation that the *H19* DMR acquires methylation from the sperm and maintains in preimplantation embryos (separated into ICM and TE) and in somatic tissues. The *MSCT2* DMR shows the opposite profile with sperm devoid of methylation. (**B**) The bisulphite PCR profiles for the novel ubiquitous *FANCC* and *SVOPL* DMRs in human sperm, blastocysts and placenta. (**C**) Methyl-seq datasets reveal that the *R3HCC1* gene has two adjacent gDMRs, an upstream paternal gDMR (region 1) that subsequently gains methylation on both alleles during the blastocyst stage and a placenta-specific maternally methylated promoter region (region 2). The vertical black lines in the methyl-seq tracks represent the mean methylation value for individual CpG dinucleotides. Green boxes highlight the position of the gDMRs. (**D**) Confirmation of the methylation profile by bisulfite PCR and subcloning. Each circle represents a single CpG dinucleotide on a DNA strand. (•) Methylated cytosine, (o) unmethylated cytosine. Each row corresponds to an individual cloned sequence. If informative, the parental-origin of methylation is indicated. For clarity only the first 10 CpG dinucleotides are shown.

Using nested-multiplex bisulphite PCR, we confirmed the methylation profiles of four ubiquitous imprinted gDMRs (*H19*, *MCTS2*, *FANCC*, *SVOPL*) and 13 placenta-specific gDMRs in sperm and blastocysts micosurgically separated into inner cell mass (ICM) and trophectoderm (TE) (Fig 3 and S4 Fig). The *R3HCC1* loci on chromosome 8 exemplified the fate of opposing germline methylation difference as this gene has adjacent oocyte and sperm-derived gDMRs. Using bisulphite PCR we show that the maternally methylated gDMR is observed in ICM/TE and term placenta, whereas the paternally methylated gDMR, which was not identified in our initial genome-wide screen since it does not reach our screening criteria having < 25 CpGs, resolves to a mosaic methylated state at the blastocysts stage (Fig 3C and 3D).

### Placenta-specific gDMR are largely confined to primates

We have previously shown that 14 orthologs of maternally methylated placenta-specific DMRs are devoid of methylation in the mouse placenta [10]. Using methyl-seq datasets from mouse placenta with bisulphite PCR confirmation we show that no human placenta-specific DMRs are conserved in mice (S7A and S7B Fig). Similarly the mouse orthologous regions corresponding to the *SVOPL* and *FANCC* DMRs also lack allelic methylation and are biallelically expressed in multiple tissues (S7B Fig). Several studies have shown that maternally methylated gDMRs mark different loci in mouse compared to humans [3, 5], suggesting that the mouse genome may possess a unique set of placenta-specific DMRs inherited from the female germline. We therefore determined the fate of oocyte-derived gDMRs in hybrid mouse placenta. Consistent with our previous observation, no maternal gDMRs persist as placenta-specific DMRs, reinforcing that this phenomenon is not observed in mice (S7C Fig). Recently methyl-seq datasets have been produced from different mammalian species, including rhesus macaque, horse, cow and dog [23]. Similar to mouse, the orthologues of the vast majority of human placenta-specific gDMRs do not have a methylation profile consistent with imprinting in non-primate species (S7A Fig). Using bisulphite PCR on DNA derived from rhesus placenta, we confirm evolutionary conservation of 63% placenta-specific DMRs as well as those associated with the ubiquitously imprinted *MCTS2*, *GRB10* and *L3MBTL1* genes (S7D Fig).

### Allele-specific expression analysis of transcripts associated with placenta-specific gDMRs

**Fig 4:**
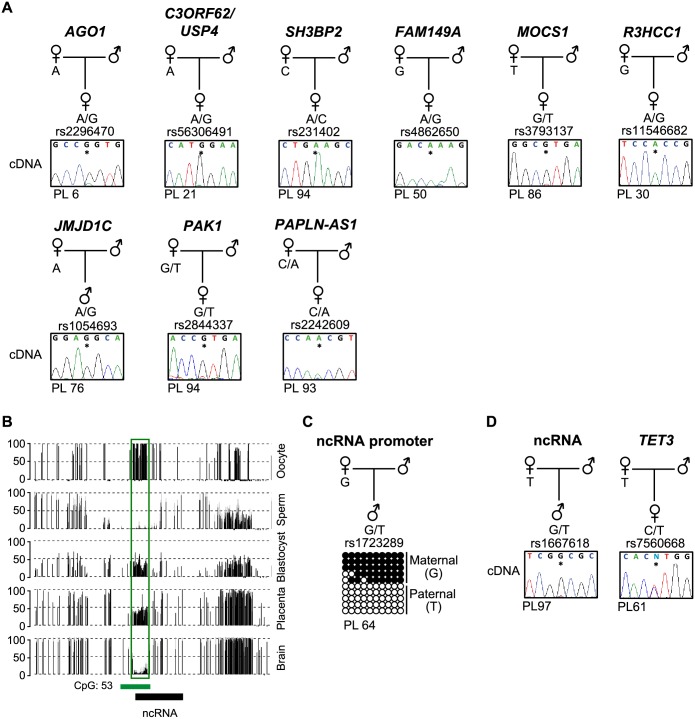
Analysis of allelic expression for genes associated with novel placenta-specific maternally methylated gDMRs. (**A**) Allelic RT-PCR analysis for nine transcripts originating from placenta-specific DMRs in control placenta samples. Monoallelic paternal expression was observed in heterozygous placenta biopsies. (**B**) The identification of a ~10 kb ncRNA overlapping a placenta-specific gDMR ~12 kb downstream of the *TET3* gene. The vertical black lines in the methyl-seq tracks represent the mean methylation value for individual CpG dinucleotides. The green box highlights the position of the gDMRs. (**C**) The 2.7 kb maternally methylated placenta-specific DMR identified by methyl-seq and confirmed with allelic-specific bisulphite PCR and subcloning. Each circle represents a single CpG dinucleotide on a DNA strand. (•) Methylated cytosine, (o) unmethylated cytosine. Each row corresponds to an individual cloned sequence. For clarity only the first 10 CpG dinucleotides are shown. (**D**) Paternal expression of the RNA-seq peak was determined by RT-PCR, whilst allele-specific RT-PCR revealed that *TET3* is biallelically expressed in term placenta samples.

**Fig 5:**
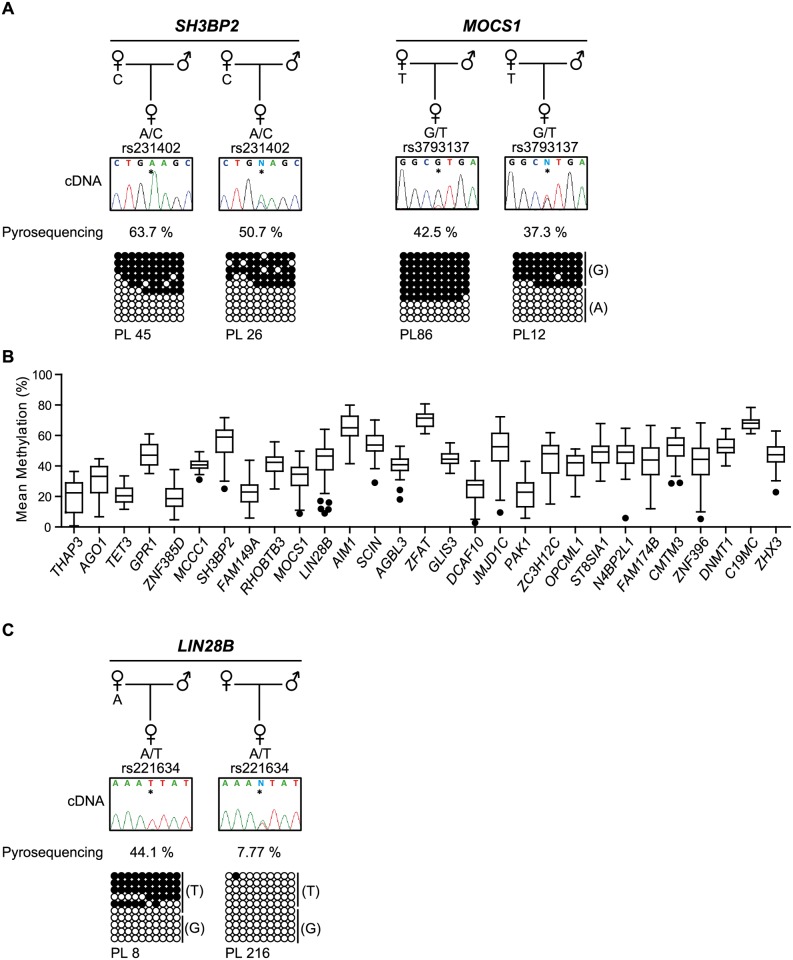
Allele-specific expression and methylation analysis of genes with variable maternal placenta-specific gDMRs. (**A**) Allelic RT-PCR analysis for *SH3BP2* and *MOCS1* in placenta samples with bisulphite PCR and subcloning of the associated gDMR in the same biopsy. Each circle represents a single CpG dinucleotide on a DNA strand, a methylated cytosine (•) or an unmethylated cytosine (o). For clarity only the first 10 CpG dinucleotides are shown. (**B**) Pyrosequencing quantification of 29 placenta-specific DMRs reveals hypomethylation indicative of a stochastic trait. The average methylation of 55 controls placenta samples from uncomplicated pregnancies reveals profiles consistent with one methylated and unmethylated allele. The controls represented as Tukey box-and-whisker plots with whiskers spanning from 25th to 75th percentiles +/- 1.5IQR to highlight outliers. Individual hypomethylated samples are highlighted. (**C**) Allelic specific RT-PCR and strand-specific bisulphite PCR and subcloning of placenta samples lacking maternal methylation at *LIN28B* identified in (**B**) compared to a normal imprinted control sample.

The main biological significance of promoter methylation is thought to be transcriptional repression of tissue-specific genes, with methylation levels negatively correlated with expression following genome activation at the 8 cell stage [6]. To determine if maternal-specific methylation at placenta-specific gDMRs dictates paternal expression we performed allele-specific RT-PCR in placenta. Paternal expression was confirmed for nine genes including *AGO1*, *USP4*, *SH3BP2*, *FAM149A*, *MOCS1*, *R3HCC1*, *JMJD1C*, *PAK1* and *PAPLN-AS* (Fig 4A; S4 Table). Curiously however, we observe that not all informative placenta samples exhibited monoallelic expression despite maintaining robust maternal methylation (Fig 5A, S4 and S8 Figs; S4 Table). We also observe paternal expression of a ~10 kb non-coding (nc)RNA overlapping a placenta-specific gDMR located 12 kb 3’ to *TET3* (Fig 4B–4D). To determine if this ncRNA influences expression *in cis*, we performed allelic RT-PCR for *TET3*. We observe biallelic expression of *TET3* suggesting that the neighboring ncRNA does not possess enhancer or repressive function in term placenta (Fig 4D). In total this bringing the total number of confirmed placenta-specific paternally expressed genes to more than 30 [8, 16].

### Widespread polymorphic maternal methylation in placenta

Polymorphic imprinting has been described for only a few loci in humans, including the *IGF2R* [24, 25] and nc886/vtRNA2-1 [26, 27], with the latter consistent with being a metastable epiallele. To determine if the placenta-specific gDMRs that we identified show variable methylation on the maternal allele, we performed pyrosequencing to quantify a larger cohort of normal placenta samples from uncomplicated pregnancies. We identified hypomethylated samples for 12 of the regions (Fig 5B), with the most affected loci being *LIN28B* and *AGBL3*. For samples with informative polymorphisms this lack of methylation is associated with biallelic expression (Fig 5C), an observation consistent with some placenta-specific maternal gDMRs being a stochastic polymorphic trait [17].

### Imprinted expression following embryonic genome activation in blastocysts

**Fig 6:**
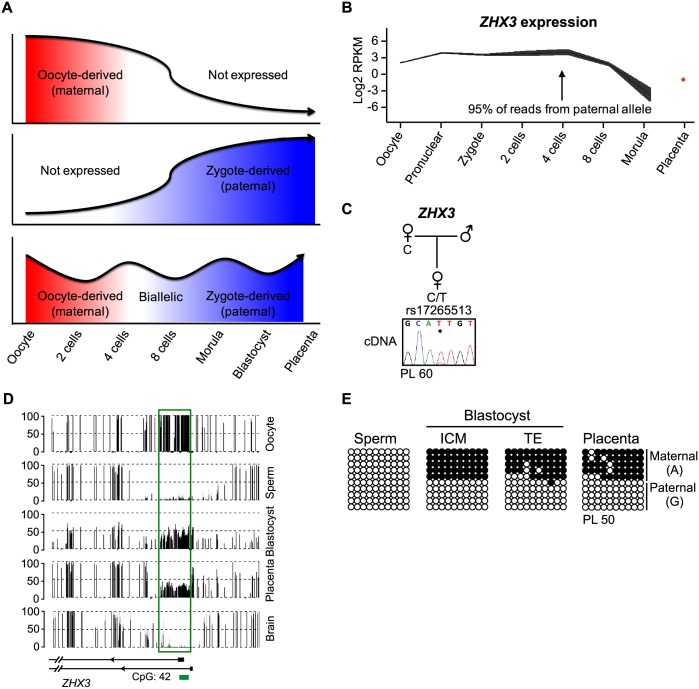
Identification of novel imprinted genes in human embryos using allele-specific RNA-seq datasets. (**A**) Schematic drawing of the sequential transcriptome switching from oocyte-derived transcripts to the embryonic genome in human preimplantation embryos. (**B**) The expression pattern of the *ZHX3* gene during human preimplantation development. High expression was observed from the zygote to the 8-cell stage, declining in the morula. Paternal expression as observed from the 4-cell stage onwards. (**C**) Allele-specific RT-PCR was performed on term placenta samples heterozygous for the exonic SNP rs17265513. (**D**) Methyl-seq traces reveal the location of the placenta-specific maternal gDMR overlapping the *ZHX3* promoter. The vertical black lines in the methyl-seq tracks represent the mean methylation value for individual CpG dinucleotides. The green box highlights the position of the gDMRs. (**E**) The methylation profile confirmed using bisulphite PCR and cloning in sperm, blastocysts and placenta. Each circle represents a single CpG dinucleotide on a DNA strand. (•) Methylated cytosine, (o) unmethylated cytosine. Each row corresponds to an individual cloned sequence. For clarity only the first 10 CpG dinucleotides are shown.

It has previously been reported that a significant proportion of transcripts are monoallelically expressed in cleavage embryo [17] indicating that maternally methylated placenta-specific gDMRs may regulate allelic expression at this earlier developmental time point. To ascertain if the placenta-specific gDMRs orchestrate imprinted expression, we determined allelic expression in publically available single cell embryo RNA-seq datasets for which paternal genotypes were available [18]. Gene expression profiles were analyzed in individual embryos to determine the progression of expression levels and their allelic origin. To compare embryos at different stages it is important to take into consideration two events, embryonic genome activation and oocyte-derived transcript degradation. Zygotic genome activation (ZGA) occurs soon after fertilization (pre-major ZGA) and processed in successive waves of activation with the major changes reported at the 4–8 cell stage [28]. Maternal transcript stores in the oocyte cytoplasm are diminished after fertilization by a combination of degradation and recruitment to the polysome and translated prior to ZGA [29]. Transcripts highly abundant at the pronuclear stage and decreasing as developmental proceeds will not be expressed from the embryonic genome and will appear maternally derived. Embryonically transcribed genes that maintain high expression levels from the pronuclear stages would appear maternally expressed before 8-cell stage, switching to imprinted paternal expression with RNA synthesis from the unmethylated allele if the gDMRs are functional. Some instances of biallelic expression maybe wrongly classified since embryonic paternal expression and oocyte-derived transcripts may co-exist until late cleavage stage. Finally, genes that are activated during cleavage embryo development, but not originally expressed in the zygote are predicted to be from the paternal allele. Therefore functional paternal expression can only be categorized after genome activation (Fig 6A). Using these criteria we screened all transcripts near the oocyte-derived gDMRs for imprinting and observed, as proof of principal, the paternal expression of *ZHX3* in 8-cell and morula and confirm preferential paternal expression arising from a maternally methylated promoter in multiple term placenta biopsies (Fig 6B–6E).

### Placenta-specific gDMR are reprogrammed in primordial germ cells

In addition to the reprogramming that occurs immediately after fertilizations from which imprints are protected, reprogramming in primordial germ cells (PGCs) of the developing fetus includes all ubiquitous imprints ensuring the transmission of genetic information with the correct epigenetic profile in the gametes [30]. Recently, the methylomes of human PGCs of both sexes have been generated, which confirm that human PGCs at 7–9 weeks gestation are hypomethylated similar to those in the mouse at embryonic day 13.5 [31, 32]. Using these datasets, we confirm that placenta-specific DMRs are devoid of methylation in both male and female PGCs at 10 weeks gestation and are indistinguishable from ubiquitous gDMR imprints (S5 Table). Similar to the ubiquitous gDMR imprints, the majority of placenta-specific gDMRs (78%) are frequently associated with CpG-rich sequences with an intragenic location with evidence of a transcriptional event initiating from upstream promoters (S5 Table). This intragenic location has been shown to be important in facilitating the acquisition of methylation during female germline development [33, 34].

## Discussion

In this study DNA methylation in human gametes, embryos, placenta and multiple somatic tissues were used to identify gDMRs that may act as imprints. Using high-density methylation arrays, our group and others have recently identified ~150 maternally methylated DMRs in placenta [10, 15–17, 35], for which we confirm the majority are *bona fide* germline difference in methylation. A comparison of the oocyte-derived DMRs reported by Smith and colleagues revealed largely overlapping datasets in blastocysts [7]. Using different bioinformatics criteria, 25 continuously CpGs rather than 100bp tiles, our analysis identified ~64% of previously identified loci, with missing regions possible due to inferior sequence coverage of reduced representation bisulphite sequencing or the size of the windows analyzed. Furthermore, using methyl-seq datasets, we identify an additional 551 loci that could represent placenta-specific gDMRs, however only 11% had high informative polymorphisms to allow for allelic discrimination. With the exception of only four regions, these placenta-specific gDMRs are associated with CpG islands or promoter intervals devoid of methylation in somatic tissues. Those regions fulfilling our criteria of partially methylation and hypermethylated in other tissues may simply reflect the relatively hypomethylated nature of the placenta genome that had previously hindered us from performing imprinted DMR analyses in placenta methyl-seq datasets [10]. Recently, Schroeder and colleagues described that the placenta genome has unique partially methylated domains (PMDs) that are larger (>100 kb) and have lower levels of DNA methylation than the rest of the genome, which are stable throughout gestation [21, 36]. The placenta-specific gDMRs we describe are much smaller than PMDs having an average size of 2.2 kb with only two (*CACNA1I* and *ZNF385D*) mapping to PMDs.

While allelic DNA methylation at ubiquitous gDMR imprints is associated with monoallelic expression, our analysis reveals that only half of all placenta-specific gDMRs orchestrate paternal expression suggesting that despite being maternally methylated, the maternal alleles may not be associated with a compact chromatin state or decorated with repressive histone modifications sufficient to influence transcription. A recent genome-wide screen using diandric and digynic triploid conceptions and RRBS datasets also identified placenta-specific gDMRs, many overlapping with the loci we identify [17]. However, these authors did not perform any allelic expression analyses for their candidates and so the functional relevance of this tissue-specific methylation was not addressed. Furthermore this study revealed epigenetic stochasticity for many of the placenta-specific DMRs described, similar to what we also observe for many of the regions we quantified using pyrosequencing (Fig 5). However it remains to be determined whether lack of methylation at these loci reflects a random selection of cells not maintaining methylation after embryonic reprogramming or alternatively, exposes loci that fail to establish methylation in the female germline in a polymorphic fashion.

We show that allelic methylation is present in the inner cell mass and trophectoderm of human blastocysts, revealing that 5mC is selectively protected from embryonic reprogramming and that it maintained following the first differentiation step. Furthermore, our data suggest that an additional small wave of targeted demethylation exists following implantation in cells specified for the somatic lineages that is absent during placenta differentiation. Very few studies have assessed allelic expression of imprinted genes in human embryos with only paternal expression of *IGF2*, *SNRPN* and *MEST* being previously reported [37–39]. We show that the placenta-specific gDMRs can influence allelic expression immediately following embryonic genome activation as highlighted by *ZHX3*. Unfortunately no additional paternally expressed genes were identified in the embryo datasets due to the lack of informative polymorphisms. Extrapolating this observations means that there are potentially thousands more transiently imprinted genes in the blastocysts associated with the loci which get that reprogrammed after implantation which may have a physiological role in embryonic development.

By directly assessing methylation in placenta-derived DNA from different mammalian species we observe that oocyte-derived gDMRs in placenta are largely restricted to primates, being most abundant in humans. These observations are inconsistent with recent reports that oocyte-derived methylation regulates trophoblast development in the mouse [40]. However, this study did not assess allelic methylation *per se*, but inferred it from various Dnmt3a/Dnmt3b knockout crosses. The developmental phenotype observed could be due to the deregulation of only a few genes such as the maternally expressed *Ascl2* (previously known as *Mash2*) that is regulated *in cis* by the maternally methylated ubiquitous *Kv*DMR1[41, 42]. Furthermore strand-specific bisulphite PCR of several of the proposed genes responsible for this developmental phenotype failed to identify methylation specifically on the maternal allele in mouse hybrid placenta (S9 Fig).

There are no unifying explanations of how imprinted genes evolved, but there are several theories hypothesized that underscore the importance of the placenta. The most popular theory is associated with the parental conflict and nutrient supply and demand hypothesis [43, 44]. However with the recent identification of developmentally important genes, including the FGFs that regulate trophoblast survival and placental angiogenesis [45], and key epigenetic regulators, such as JMJD1C which is involved in regulating early preimplantation development of bovine embryos [46], we favor the hypothesis that maternal silencing is a mechanism to prevent ovarian teratomas that arise from parthenogenetically activated oocytes [47, 48].

Our study has shown that oocyte-derived methylation can uniquely be maintained as DMRs in the extra-embryonic lineages, with many placenta-specific DMRs coordinating paternal expression following embryonic genome activation. Our data corroborates the observations that these placenta-specific gDMRs can be polymorphic, with a minority of samples being unmethylated [17]. It remains to be seen if the lack of these placenta-specific DMRs influences pregnancy outcomes and whether they are involved in implantation and preimplantation embryo viability.

## Materials and Methods

### Ethics statement

Ethical approval for the use of human placenta samples was granted by the Institutional Review Boards at the National Center for Child Health and Development (project 234), Hospital St Joan De Deu Ethics Committee (35/07) and Bellvitge Institute for Biomedical Research (PR006/08). The use of surplus human embryos for this study was evaluated and approved by the scientific and ethic committee of the Instituto Valenciano de Infertilidad (IVI) (1310-FIVI-131-CS), Bellvitge Institute for Biomedical Research Ethics Committee (PR292/14), the National Committtee for Human Reproduction (CNRHA) and the Regional Health Counsel of Valencia.

Mouse work was approved by the Institutional Review Board Committees at the National Center for Child Health and Development (approval number A2010-002).

A single placenta sample from rhesus macaque was obtained from the breeding colony of the Biomedical Primate Research Center, Rijswijk, Netherlands using protocols approved by the Committee on the Ethics of Animal Tissue Collection at BPRC (Permit # 730). The EUPRIM-Net Bio-Bank is conducted and supervised by the scientific government board along all lines of EU regulations and in harmonization with Directive 2010/63/EU on the Protection of Animals Used for Scientific Purposes.

### Biological samples

A cohort of 72 human term placenta biopsies (gestational age 35–41 weeks gestation, average 37 weeks) from uncomplicated pregnancies with their corresponding maternal blood samples were collected at Hospital St Joan De Deu (Barcelona, Spain) and the National Center for Child Health and Development (Tokyo, Japan). Written informed consent was obtained from all participants. All placenta biopsies were collected from the fetal side around the cord insertion site. The placenta-derived DNA samples were free of maternal DNA contamination based on microsatellite repeat analysis. Both DNA and RNA extractions and cDNA synthesis were carried out as previously described [22].

Three surplus human blastocysts were recruited at the Fundación Instituto Valenciano de Infertilidad (FIVI) in Valencia. The blastocysts were thawed using the Cryotop method following manufacturer’s instructions [49] and incubated in CCM medium (Vitrolife, Göteborg, Sweden) for 6–12 hours before microdissection in order to allow their full expansion and the inner cell mass (ICM) and trophectoderm (TE), that were subsequently separated by micromanipulation using laser technology (OCTAX, Herborn, Germany). The separated ICMs and TEs were individually placed in PCR tubes containing 2.5 μL of PBS and immediately snap frozen at -80°C until processing.

Wild type mouse embryos and placentas were produced by crossing C57BL/6 with *Mus musculus molosinus* or *Mus musculus castaneous* mice. Animal husbandry and breeding were conducted according to the institutional guidelines for the care and the use of laboratory animals.

A single placenta sample from rhesus macaque (animal 95023) was obtained from the breeding colony of the Biomedical Primate Research Center, Rijswijk, following a C-section procedure.

### Human methyl-seq data analysis

We analysed twenty-eight publicly available methylomes obtain from GEO or NBDC repositories. Two datasets were derived from human oocytes (JGAS00000000006), 5 from human sperm (JGAS00000000006 and GSE30340), 3 from brain (GSM913595, GSM916050, GSM1134680) 3 from CD4+ lymphocytes (GSE31263), 2 from liver (GSM916049, GSM1134681) and individual datasets from preimplantation embryos (JGAS00000000006), placenta (GSM1134682), muscle (GSM1010986), CD34+ cells (GSM916052), sigmoid colon (GSM983645), lung (GSM983647), aorta (GSM983648), esophagus (GSM983649), small intestine (GSM983646), pancreas (GSM983651), spleen (GSM983652), adrenal (GSM1120325) and adipose tissue (GSM1010983). Methylation calls were mapped to the hg19 genome. CpG methylation values were calculated using reads from both strands as (methylated / (methylated + unmethylmated). Only CpGs covered by at least 5 reads were considered for the analysis. For samples with duplicates, the average of methylation was used except for oocyte samples that present a low coverage. For this sample the methylated and unmethylated calls of the two experiments were sum to calculate the methylation ratio. Using the cut off of 5 reads per CpG, the coverage of all experiment vary from 89.6% up to 96.9% of all the CpGs, except for the oocyte methylomes that cover 54.8% of CpGs sites.

### Identification of germline DMRs

The methylomes for oocyte and sperm were screen with a sliding windows approach to identify methylated and umethylated intervals. Windows were defined as 25 consecutive CpGs and was only considered if the methylation levels was present for at least 10 CpG sites. This windows was classified methylated if mean_25CpGs_—1SD_25CpGs_ > 0.75 and unmethylated if mean_25CpGs_ + 1SD_25CpGs_ < 0.25. Overlapping windows with the same classification were merge and allowed us to identify 40025 unmethylated (Us) and 177787 methylated (Ms) region in sperm and 118853 unmethylated (Uo) and 102858 methylated (Mo) regions in oocyte. A germline DMR was identify when opposite methylated regions in sperm and oocyte overlap for more than 25 CpGs and the position defined by the overlapping difference between methylated regions in sperm and oocyte.

### Identification of partially methylated regions

Intermediately methylated region in blastocysts, placenta and somatic tissues were identify using the sliding windows approach with the following criteria 0.2 < mean_25CpGs_ +/- 1.5SD_25CpGs_ < 0.8. Consecutive windows on each sample were fused to generate only a single region. A gDMR was considered to be conserved in preimplantation embryo if the gDMR overlap with a partially methylated region in the blastocyst dataset. To identify the gDMR that persist in somatic tissues, all partially methylated region obtain in the 15 tissues were merge and the number of samples partially methylated for each region is attribute to each region. Only regions > 500 bp were considered to generate the partial methylation region in tissues. To be considered as a ubiquitous gDMR, the partially methylated regions have to persist in the blastocyst and in at least 12 somatic tissues. Placenta-specific gDMR were identified when the partially methylated region is conserved at blastocyst stage but is not observed in additional tissues methylomes. All positional annotations (CpG islands, repeats and gene locations, etc) were obtained from UCSC web browser and genome build hg19.

### Methyl-seq analysis in other mammals

We used the methyl-seq datasets from GSE63330 that contains placenta methylation information from rhesus macaque, dog, horse, cow and mouse [23]. The orthologous genomic intervals associated with the 551 human oocyte-derived gDMR that maintained an intermediate methylation profiles throughout embryonic reprogramming and in placenta were extracted using the UCSC LiftOver function.

### Allelic RNA-seq analysis

The abundance and genotypes of highly informative exonic SNPs within the transcripts flanking the gDMRs that maintained an intermediate methylation profile in blastocysts were called using Tophat v1.4.0 [50] (for the alignment) and Samtools v1.2 [51] (for the filtering and allelic count) in two published single cell RNA-seq datasets for preimplantation embryos (GSE44183 [18]; GSE36552 [28]). For the purpose of this study the data from individual cells were merged to reconstruct each embryo. In the case of the GSE44183 dataset the embryonic genotypes were compared to the accompanying paternal exome-seq data from the sperm donor’s blood sample.

### Genotyping and imprinting analysis

Genotypes of potential SNPs identified in the UCSC hg19 browser were obtained by PCR and direct sequencing. Sequence traces were interrogated using Sequencher v4.6 (Gene Codes Corporation, MI) to distinguish heterozygous and homozygous samples. Heterozygous sample sets were analyzed for either allelic expression using RT-PCR, methylation-sensitive genotyping or bisulphite PCR, incorporating the polymorphism within the final PCR amplicon so that parental alleles could be distinguished (for primer sequences see S6 Table).

### Methylation-sensitive genotyping

5hmC- 5 μg of heterozygous placenta DNA was subject to DNA Glucosylation using the Epimark kit (New England Biolabs) and the DNA subject to digestion with 100 units of *Msp*1 for a minimum of 8 hours at 37°C. The DNA was subject to proteinase K digestion prior to PCR.

5mC- Approximately 1 μg of heterozygous placenta DNA was digested with 10 units of *Hpa*II restriction endonuclease for 6 hours at 37°C. The digested DNA was subject to ethanol precipitation and resuspended in a final volume of 20 μl TE. Approximately 50 ng of digested DNA was used in each amplification reaction using Bioline Taq polymerase for 35–40 cycles (for primer sequences see S6 Table). The resulting amplicons were sequenced and the sequences traces compared to those obtained for the corresponding undigested DNA template.

### Bisulphite PCR of placenta-derived DNA

For standard bisulphite conversion approximately 1 μg DNA was subjected to sodium bisulphite treatment and purified using the EZ DNA methylation-Gold kit (ZYMO, Orange, CA). Approximately 2 ul of bisulphite converted DNA was used in each amplification reaction using Immolase Taq polymerase (Bioline) at 45 cycles and the resulting PCR product cloned into pGEM-T easy vector (Promega) for subsequent subcloning and sequencing (for primer sequence see S6 Table).

### Nested-multiplex bisulphite PCR of embryos and oocytes

Surgically separated ICM and TE biopsies were subject to bisulphite conversion using the EZ DNA Methylation-Direct kit (ZYMO, Orange, CA). We employed a multiplex nest PCR approach to maximize data generation. Two sets of primers were designed to each locus and robustly optimized in placenta-derived bisulphite DNA to ensure efficient amplification of both methylated and unmethylated strands at a single annealing temperature without contamination or the formation of primer dimer.

All subsequent outer primers (for ~20 separate loci) were co-amplified in the first reaction using Immolase Taq polymerase (Bioline) for 45 cycles. Second round of amplifications specific to each region, also 45 cycles, utilized locus-specific inner primers using 1ul of first round PCR as template. All second round nested PCR products were subcloned into pGEM-T easy vector for direct sequencing (for primer sequence see S6 Table).

### Pyrosequencing analysis for methylation quantification

Approximately 50 ng of bisulphite converted DNA was used for pyrosequencing following previously described protocols [22]. Standard bisulphite PCR was used to amplify the imprinted DMRs with the exception that one primer was biotinylated (for primer sequences see S6 Table). For sequencing, forward primers were designed to the complementary strand. The pyrosequencing reaction was carried out on a PyroMark Q96 instrument. The peak heights were determined using Pyro Q-CpG1.0.9 software (Biotage).

## Supporting Information

S1 Fig Analysis of candidate ubiquitous maternally methylated gDMRs. Strand-specific bisulphite PCR for *PTCHD3*, *LINC00434*, *NPAS3*, *GPR78*, *JAKMIP1*and *A1BG-AS1* in various tissues reveals inconsistent allelic methylation profiles.
(PDF)

S2 Fig Analysis of allelic methylation using *Hpa*II-sensitive (5mC) and *Msp*1-glucosylated (5hmC) genotyping. (**A**) Representative gel electrophoresis of PCR amplicons targeting placenta-specific DMRs of *TMEM17*, *FRDM3* and *KCNQ1* distinguishing 5mC from 5hmC. In all cases the resulting methylation was 5mC not 5hmC. (**B**) The sequence traces of PCR products generated using *Hpa*II digested DNA in heterozygous placenta samples.
(PDF)

S3 Fig Methylation profiles as determined by methylation-sensitive genotyping. The sequence traces of PCR products generated using *Hpa*II digested DNA (CCGG) reveals widespread maternal methylation of 21 loci in placenta samples.
(PDF)

S4 Fig Strand-specific bisulphite PCR and sequencing of novel placenta-specific gDMRs in human samples. (**A**) Confirmation of the strand-specific and allelic methylation of 12 candidate DMRs by bisulphite PCR and subcloning in sperm, preimplantation embryos (separated into ICM and TE) and term placenta biopsies. (**B**) Confirmation of placenta-specific DMR status for a further 11 regions. Each circle represents a single CpG dinucleo- tide on a DNA strand, a methylated cytosine (•) or an unmethylated cytosine (o) with the letters in the parentheses indicating SNP genotype. For clarity only the first 10 CpG dinucleotides are shown.
(PDF)

S5 Fig Characterization of the four maternally methylated placenta-specific gDMRs associated with loci that become hypermethylated in somatic tissues. The promoters of the *TMEM247*, *GPR1-AS*, *ZFAT* and *C19MC* miRNA cluster exhibit promoters that are unmethylated in sperm, hypermethylated in oocytes and intermediate methylation in blastocysts and placenta but are hypermethylated in liver (as an example of the 14 somatic tissues analyzed). The vertical black lines in the methyl-seq tracks represent the mean methylation value for individual CpG dinucleotides. The green boxes highlight the position of the gDMRs. Bisulphite PCRs on placenta derived-DNA were used for confirmation. Each circle represents a single CpG dinucleotide on a DNA strand. (•) Methylated cytosine, (o) unmethylated cytosine. Each row corresponds to an individual cloned sequence. If informative, the parental-origin of methylation is indicated. For clarity only the first 10 CpG dinucleotides are shown.
(PDF)

S6 Fig Strand-specific bisulphite PCR and sequencing of false-positive loci. (**A**) The sequence traces of PCR products generated using *Hpa*II digested DNA (CCGG) reveals that regions that appeared maternally methylated in placenta and hypermethylated in other somatic tissues in methyl-seq analysis are often false-positives regions (**B**) Bisulphite PCR and subcloning showing biallelic or mosaic methylation profile for the above regions. Each circle represents a single CpG dinucleotide on a DNA strand. (•) Methylated cytosine, (o) unmethylated cytosine. Each row corresponds to an individual cloned sequence. If informative, the parental-origin of methylation is indicated. For clarity only the first 10 CpG dinucleotides are shown.
(PDF)

S7 Fig Analysis of the orthologous sequences associated with human placenta-specific DMRs in different mammalian species. (**A**) A heatmap showing the methylation profiles of human placenta-specific gDMRs in methyl-seq datasets from placenta samples of rhesus macaque, horse, cow, dog and mouse. (**B**) The allelic methylation of the murine orthologous loci of human gDMRs using placenta DNA from intersubspecific mouse crosses (C57BL6/J—B–with JF1 –J—or *Mus musculus castaneous*–C–. Allelic RT-PCR examples of bialleic expression of *Fancc* and *Svopl* in mouse tissues. (**C**) Strand-specific methylation analysis in DNA-derived from mouse BxJF1 placenta samples for mouse gDMRs that maintain as partially methylated regions in mouse placenta methyl-seq datasets. (**D**) The allelic methylation of the rhesus macaque orthologous loci of human gDMRs. Each circle represents a single CpG dinucleotide on a DNA strand, a methylated cytosine (•) or an unmethylated cytosine (o). For clarity only the first 10 CpG dinucleotides are shown.
(PDF)

S8 Fig Analysis of allelic expression for genes associated with novel placenta-specific maternally methylated gDMRs. Allelic RT-PCR analysis for eight genes located near placenta-specific gDMRs in control placenta samples. Robust biallelic expression was observed in heterozygous placenta biopsies.
(PDF)

S9 Fig Placenta methylation profiling of regions proposed by Branco and colleagues to influence trophoblast development. Strand-specific methylation analysis in DNA-derived from mouse BxC placenta samples for the promoter intervals associated with the *Stk10*, *Scml2* and *Spry1* genes.
(PDF)

S1 Table Tissue methyl-seq profiles of the known and candidate ubiquitous maternally methylated gDMR regions. (XLSX)

S2 Table Tissue methyl-seq profiles of oocytes-derived and sperm-derived gDMRs maintaining intermediate methylation in blastocysts and placenta. The size of the placenta-specific DMRs in tabs 2 and 4 correspond to those defined by the placenta methyl-seq.
(XLSX)

S3 Table The number of heterozygous placenta samples used to determine allelic methylation. (DOCX)

S4 Table The number of heterozygous placenta samples used to determine allelic expression. (DOCX)

S5 Table Methyl-seq profiles for ubiquitous and placenta-specific gDMRs in both male and female PGCs datasets. (XLSX)

S6 Table A list of all primer sequence used in this study. (XLSX)
