# Supplementary figures and images for: Human Oocyte-Derived Methylation Differences Persist in the Placenta Revealing Widespread Transient Imprinting
Source: PLoS Genet. 2016 Nov 11;12(11):e1006427. doi: 10.1371/journal.pgen.1006427 (PMC5106035; doi:10.1371/journal.pgen.1006427)

S1\_Fig

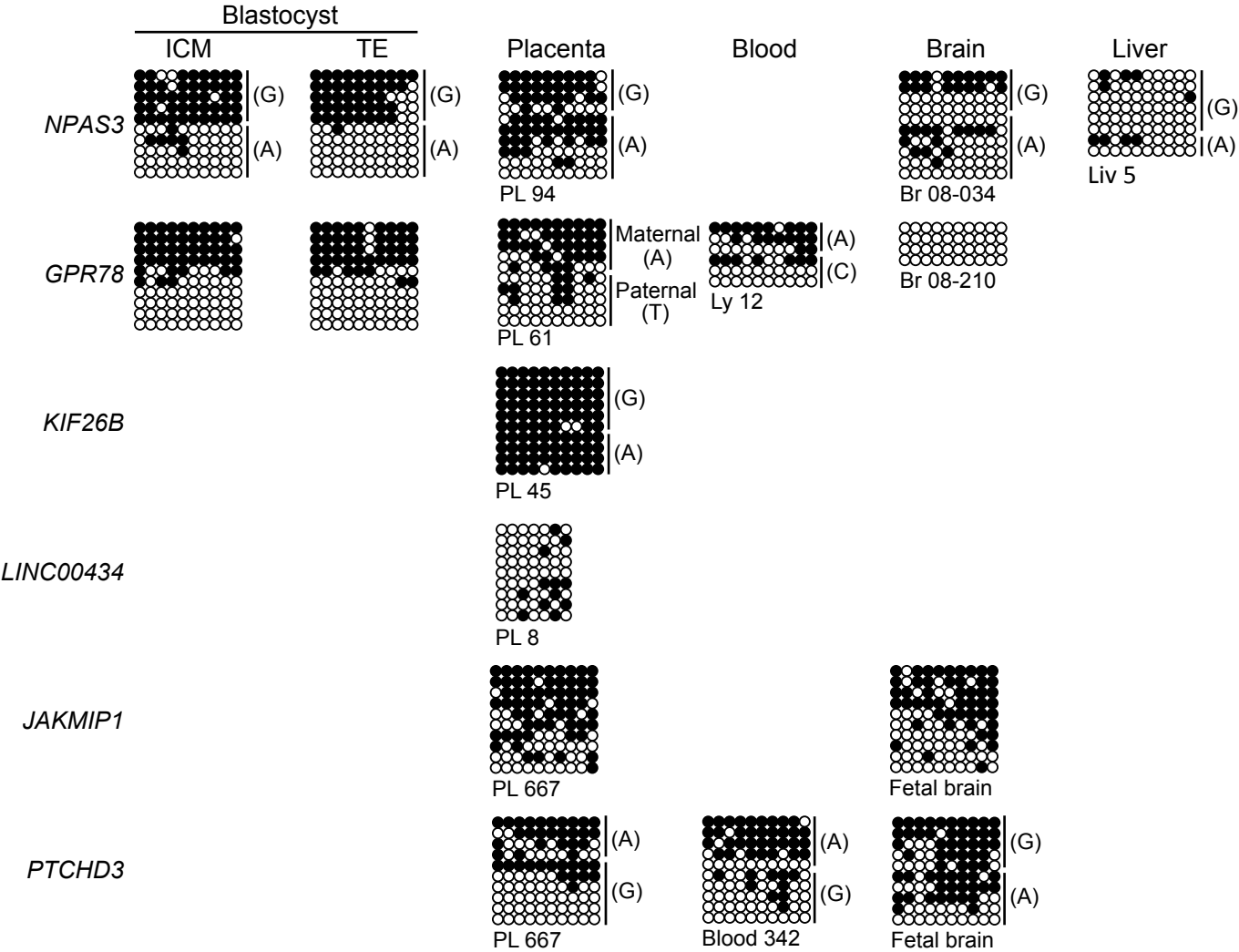

Supplement: S1 Fig — Strand-specific bisulphite PCR for PTCHD3, LINC00434, NPAS3, GPR78, JAKMIP1and A1BG-AS1 in various tissues reveals inconsistent allelic methylation profiles. (PDF) [file pgen.1006427.s001.pdf]

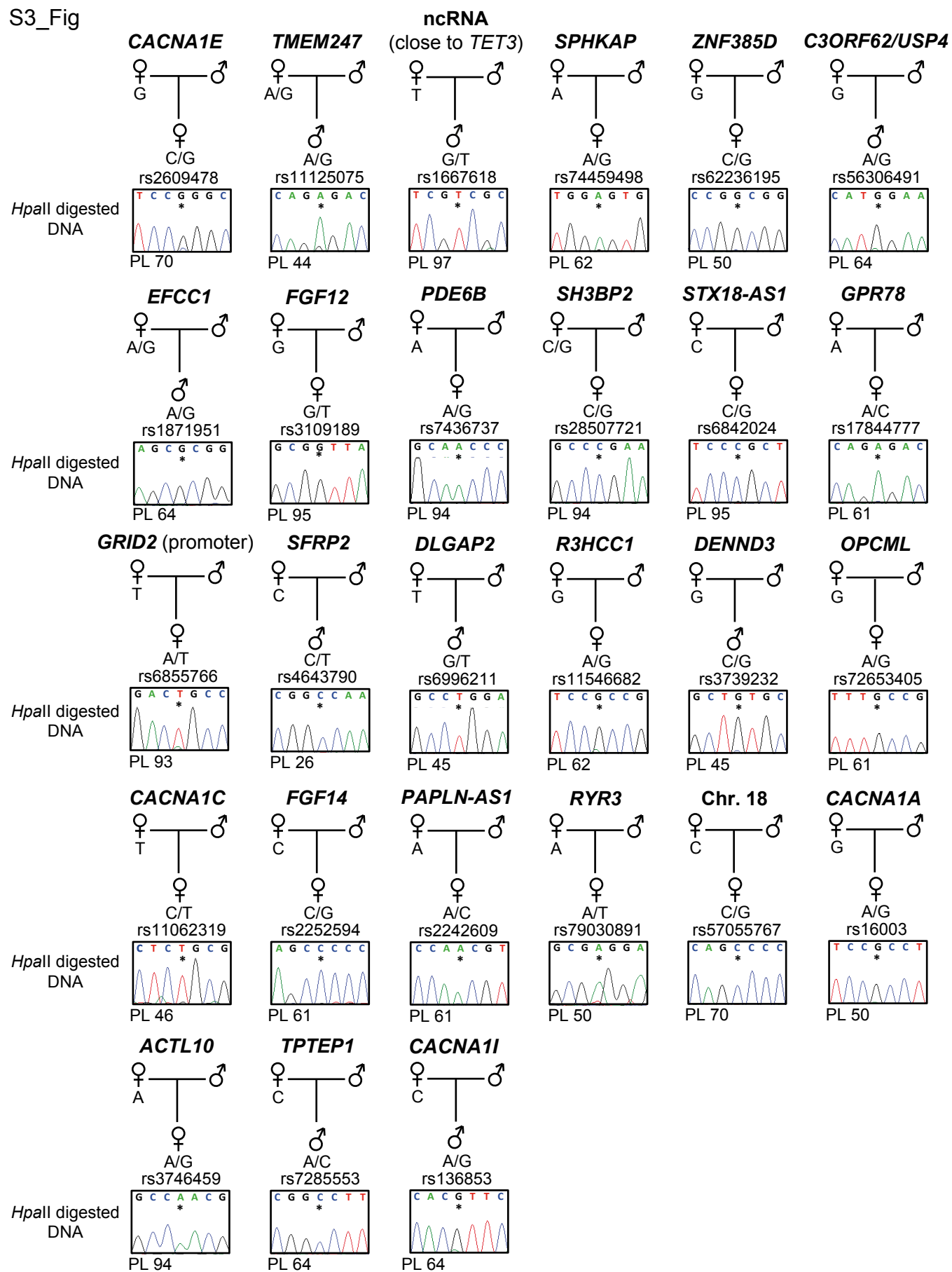

Supplement: S3 Fig — The sequence traces of PCR products generated using HpaII digested DNA (CCGG) reveals widespread maternal methylation of 21 loci in placenta samples. (PDF) [file pgen.1006427.s003.pdf]

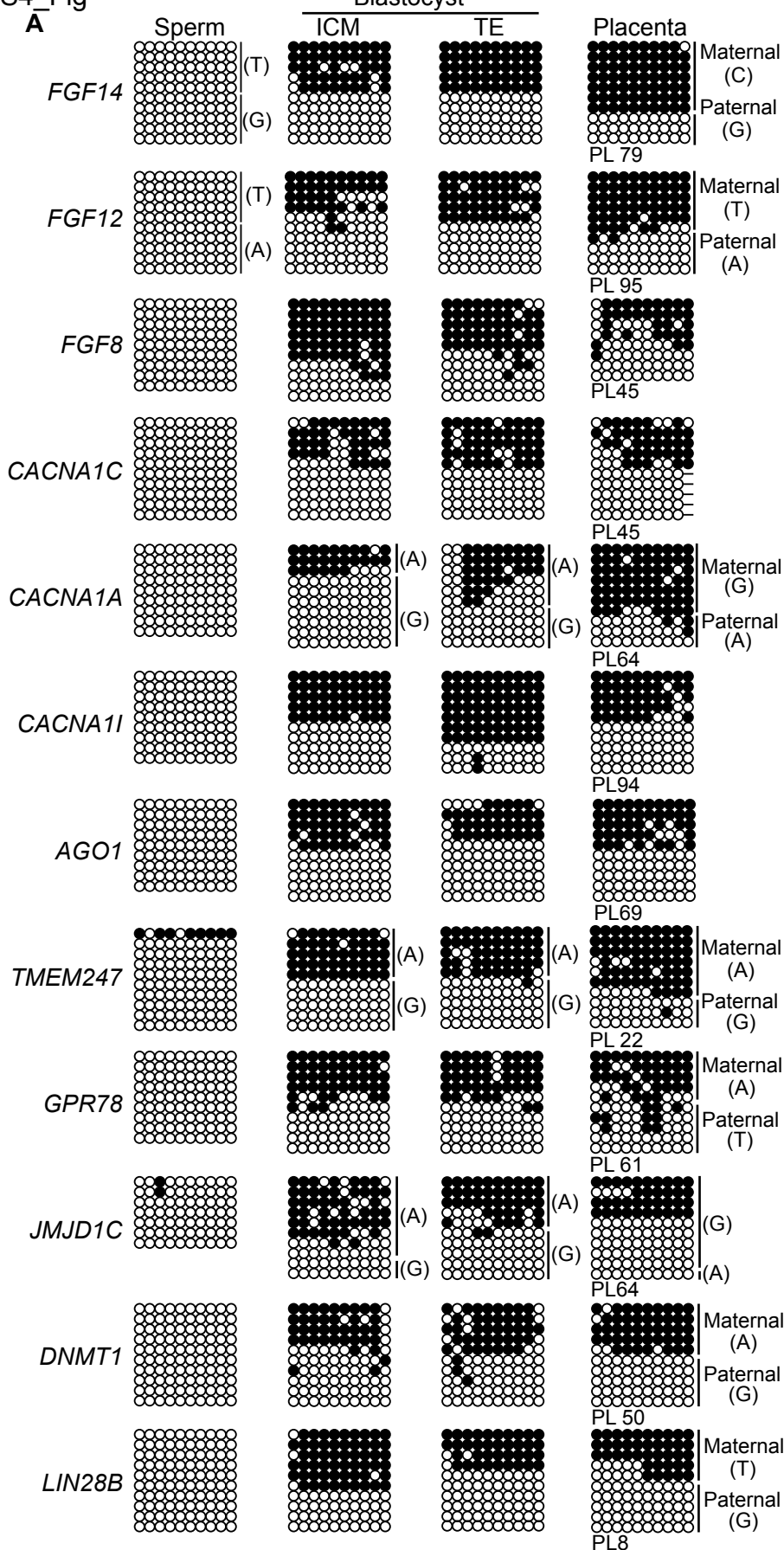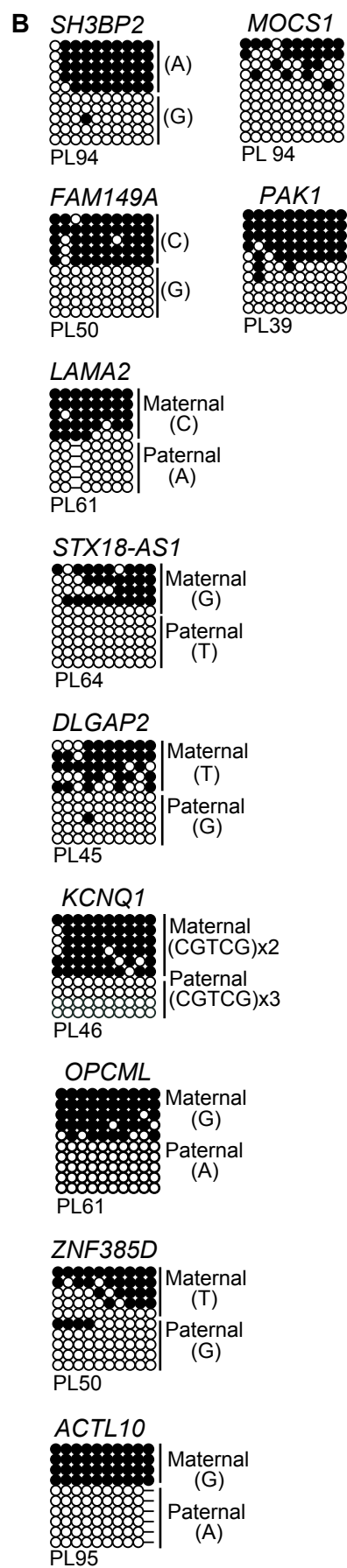

Supplement: S4 Fig — (A) Confirmation of the strand-specific and allelic methylation of 12 candidate DMRs by bisulphite PCR and subcloning in sperm, preimplantation embryos (separated into ICM and TE) and term placenta biopsies. (B) Confirmation of placenta-specific DMR status for a further 11 regions. Each circle represents a single CpG dinucleo- tide on a DNA strand, a methylated cytosine (•) or an unmethylated cytosine (o) with the letters in the parentheses indicating SNP genotype. For clarity only the first 10 CpG dinucleotides are shown. (PDF) [file pgen.1006427.s004.pdf]

**TMEM247**

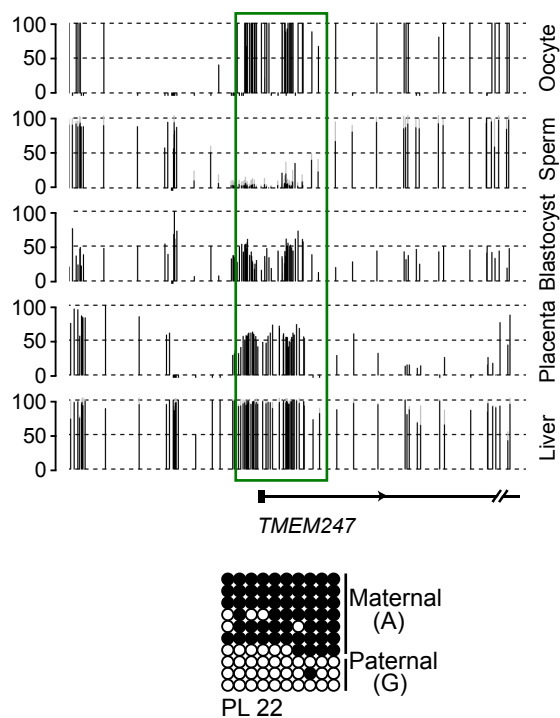

**GPR1-AS**

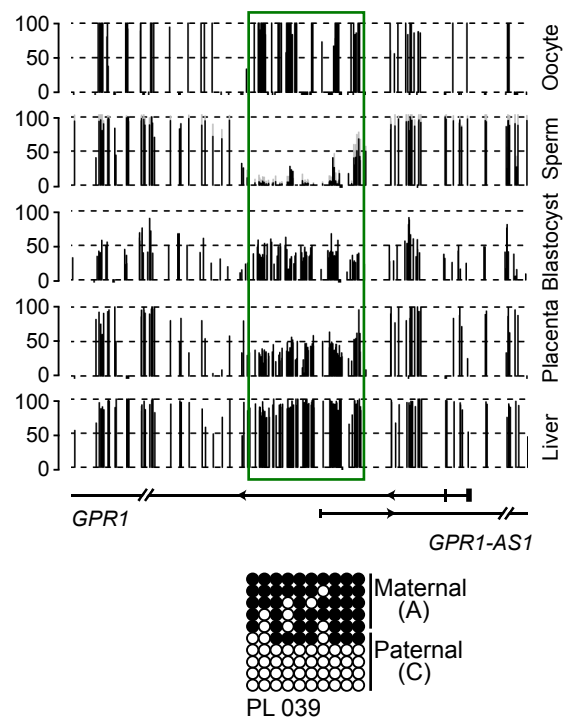

**ZFAT**

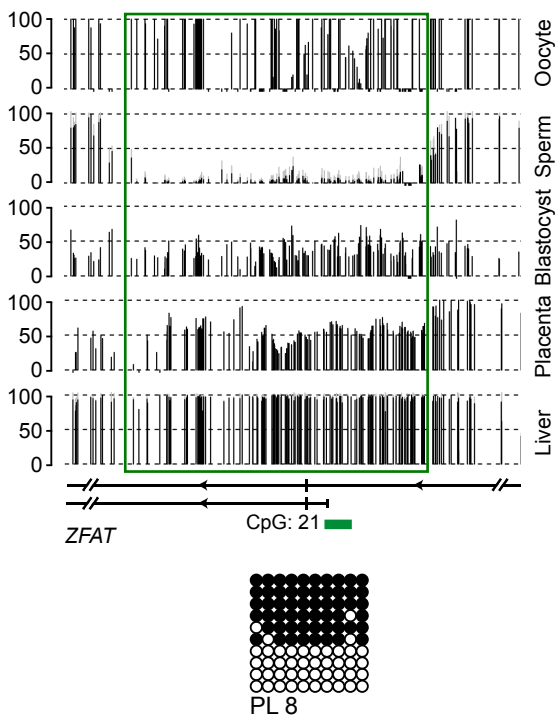

**C19MC**

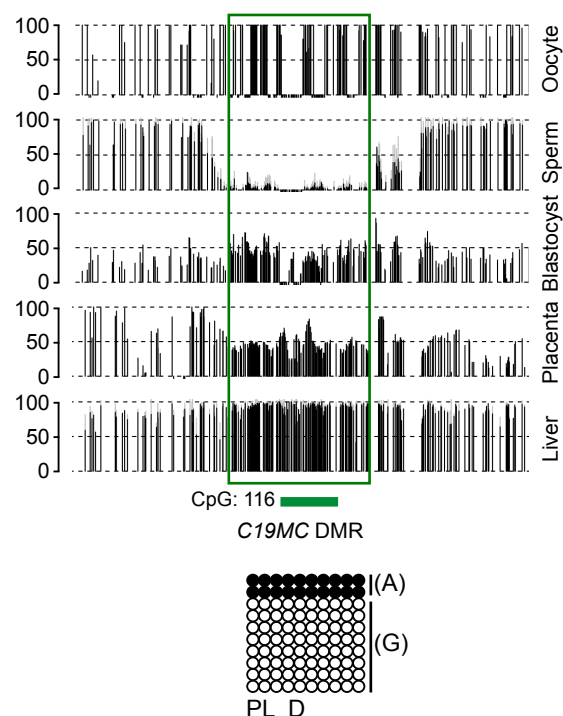

Supplement: S5 Fig — The promoters of the TMEM247, GPR1-AS, ZFAT and C19MC miRNA cluster exhibit promoters that are unmethylated in sperm, hypermethylated in oocytes and intermediate methylation in blastocysts and placenta but are hypermethylated in liver (as an example of the 14 somatic tissues analyzed). The vertical black lines in the methyl-seq tracks represent the mean methylation value for individual CpG dinucleotides. The green boxes highlight the position of the gDMRs. Bisulphite PCRs on placenta derived-DNA were used for confirmation. Each circle represents a single CpG dinucleotide on a DNA strand. (•) Methylated cytosine, (o) unmethylated cytosine. Each row corresponds to an individual cloned sequence. If informative, the parental-origin of methylation is indicated. For clarity only the first 10 CpG dinucleotides are shown. (PDF) [file pgen.1006427.s005.pdf]

A

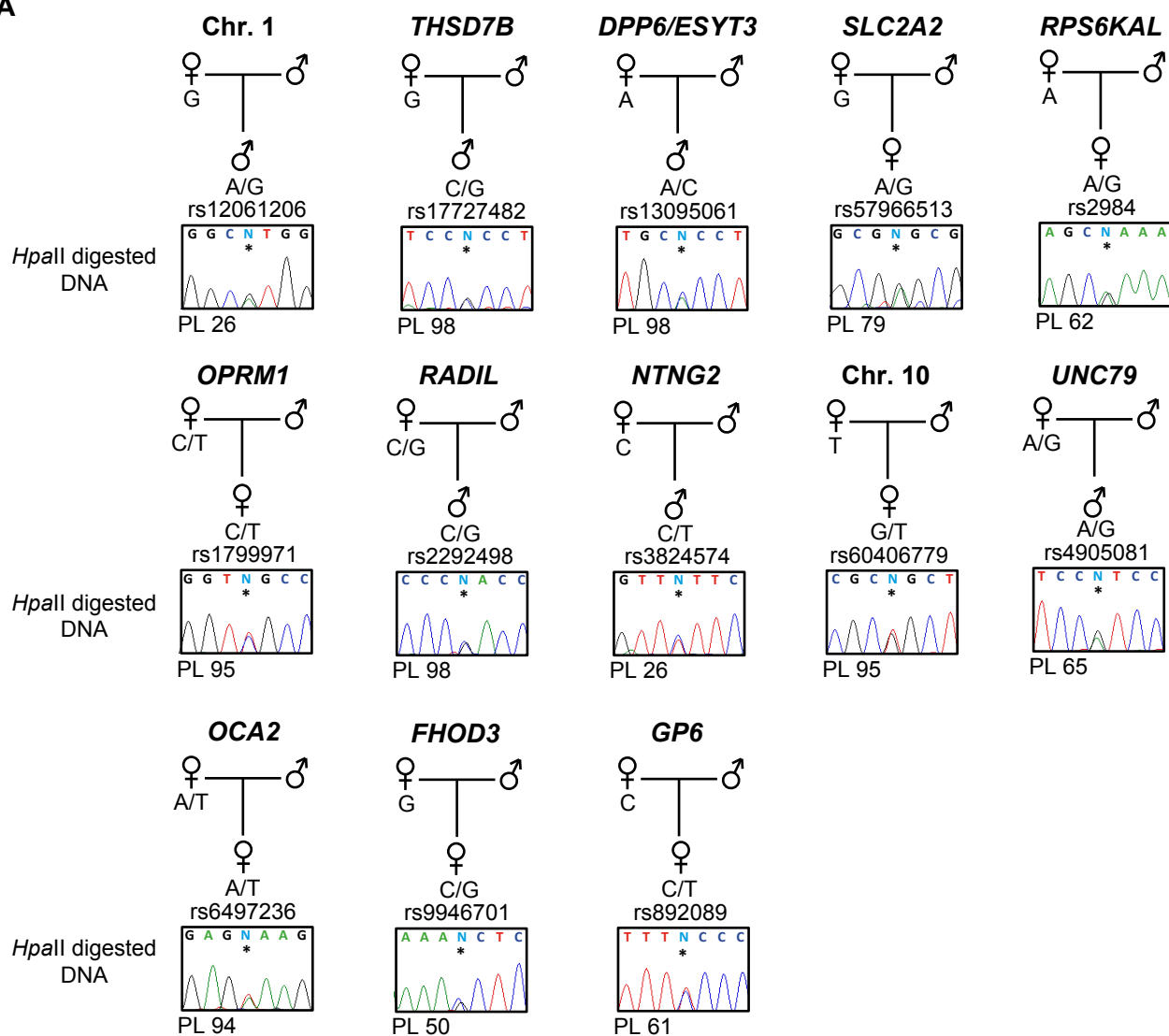

B

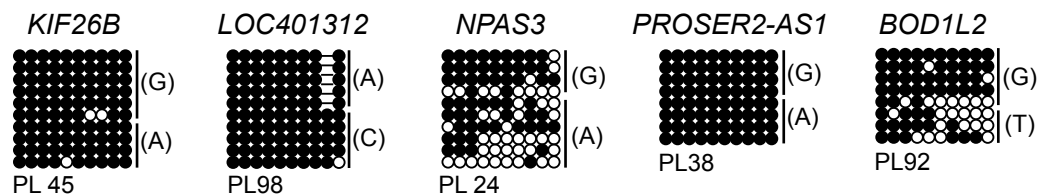

Supplement: S6 Fig — (A) The sequence traces of PCR products generated using HpaII digested DNA (CCGG) reveals that regions that appeared maternally methylated in placenta and hypermethylated in other somatic tissues in methyl-seq analysis are often false-positives regions (B) Bisulphite PCR and subcloning showing biallelic or mosaic methylation profile for the above regions. Each circle represents a single CpG dinucleotide on a DNA strand. (•) Methylated cytosine, (o) unmethylated cytosine. Each row corresponds to an individual cloned sequence. If informative, the parental-origin of methylation is indicated. For clarity only the first 10 CpG dinucleotides are shown. (PDF) [file pgen.1006427.s006.pdf]

A

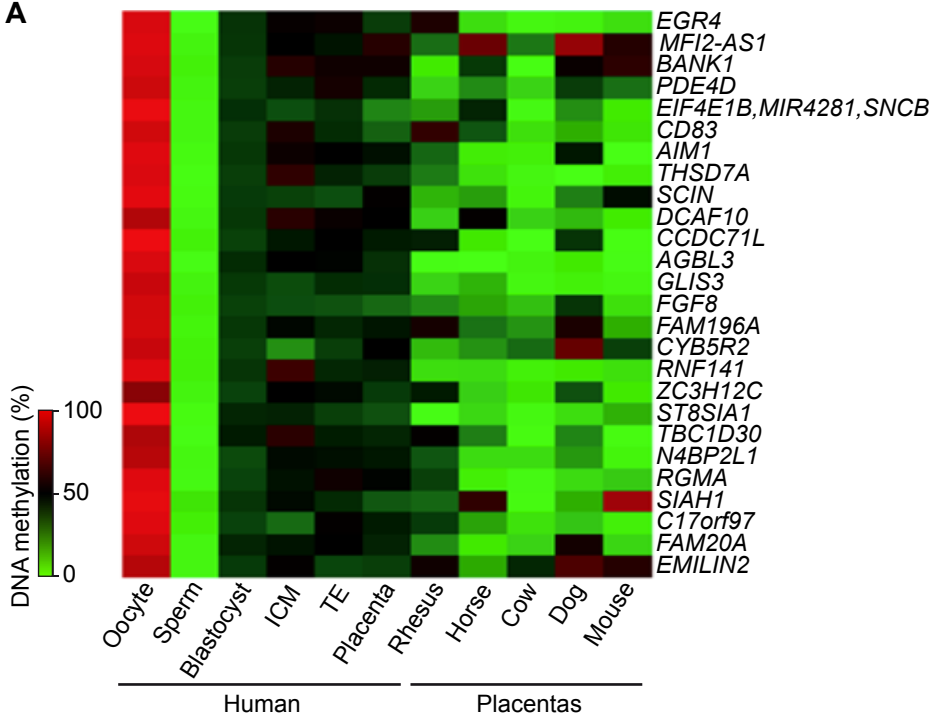

## B Human DMRs in *Mus musculus*

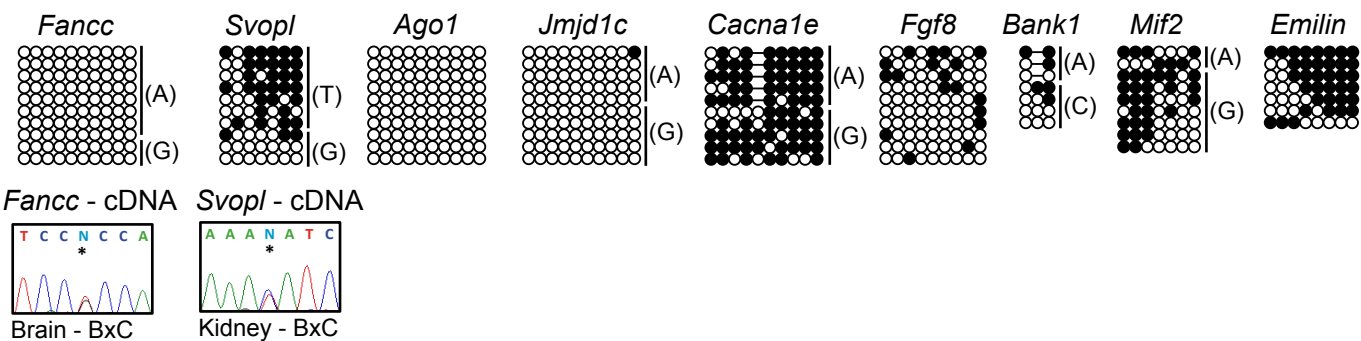

## C *Mus musculus* - Germline DMRs

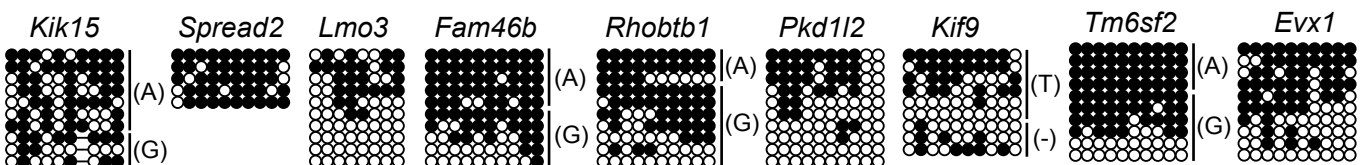

## D Human DMRs in *Macaca mulatta*

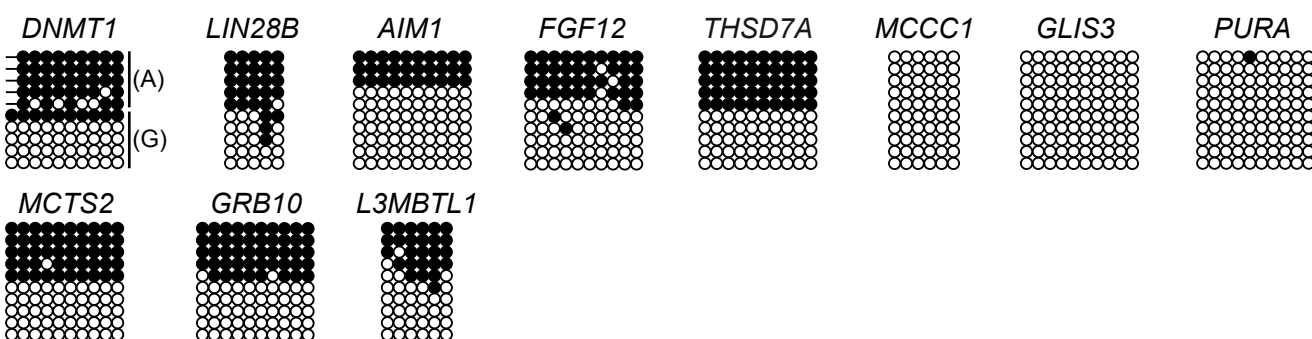

Supplement: S7 Fig — (A) A heatmap showing the methylation profiles of human placenta-specific gDMRs in methyl-seq datasets from placenta samples of rhesus macaque, horse, cow, dog and mouse. (B) The allelic methylation of the murine orthologous loci of human gDMRs using placenta DNA from intersubspecific mouse crosses (C57BL6/J—B–with JF1 –J—or Mus musculus castaneous–C–. Allelic RT-PCR examples of bialleic expression of Fancc and Svopl in mouse tissues. (C) Strand-specific methylation analysis in DNA-derived from mouse BxJF1 placenta samples for mouse gDMRs that maintain as partially methylated regions in mouse placenta methyl-seq datasets. (D) The allelic methylation of the rhesus macaque orthologous loci of human gDMRs. Each circle represents a single CpG dinucleotide on a DNA strand, a methylated cytosine (•) or an unmethylated cytosine (o). For clarity only the first 10 CpG dinucleotides are shown. (PDF) [file pgen.1006427.s007.pdf]

S8\_Fig

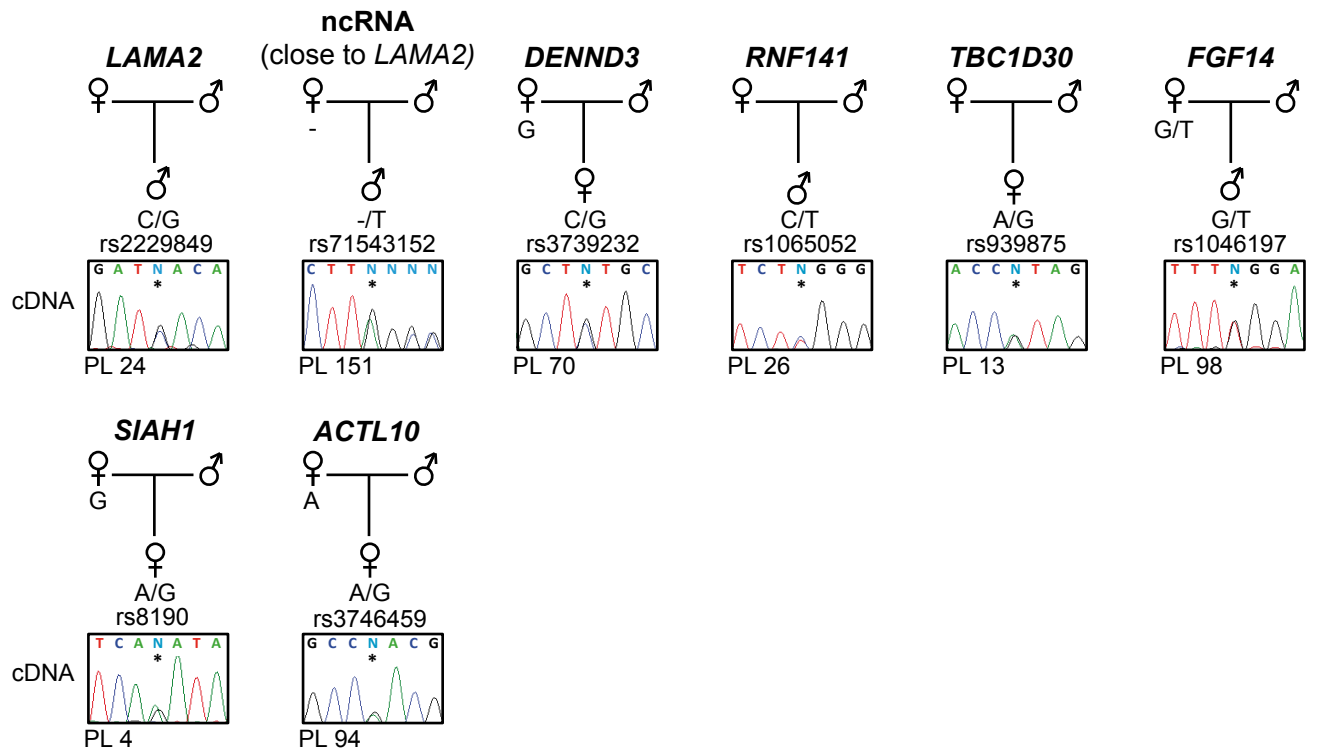

Supplement: S8 Fig — Allelic RT-PCR analysis for eight genes located near placenta-specific gDMRs in control placenta samples. Robust biallelic expression was observed in heterozygous placenta biopsies. (PDF) [file pgen.1006427.s008.pdf]

S9\_Fig

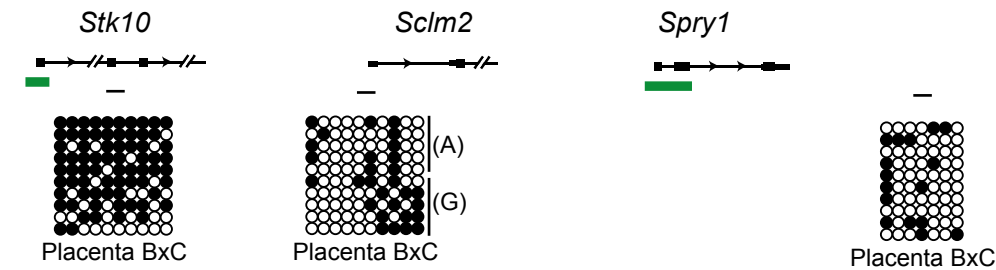

Supplement: S9 Fig — Strand-specific methylation analysis in DNA-derived from mouse BxC placenta samples for the promoter intervals associated with the Stk10, Scml2 and Spry1 genes. (PDF) [file pgen.1006427.s009.pdf]
