# Supplementary material for: Human Oocyte-Derived Methylation Differences Persist in the Placenta Revealing Widespread Transient Imprinting
Source: PLoS Genet. 2016 Nov 11;12(11):e1006427. doi: 10.1371/journal.pgen.1006427 (PMC5106035; doi:10.1371/journal.pgen.1006427)
Supplement: S2 Fig — (A) Representative gel electrophoresis of PCR amplicons targeting placenta-specific DMRs of TMEM17, FRDM3 and KCNQ1 distinguishing 5mC from 5hmC. In all cases the resulting methylation was 5mC not 5hmC. (B) The sequence traces of PCR products generated using HpaII digested DNA in heterozygous placenta samples. (PDF) [file pgen.1006427.s002.pdf]

S2_Fig

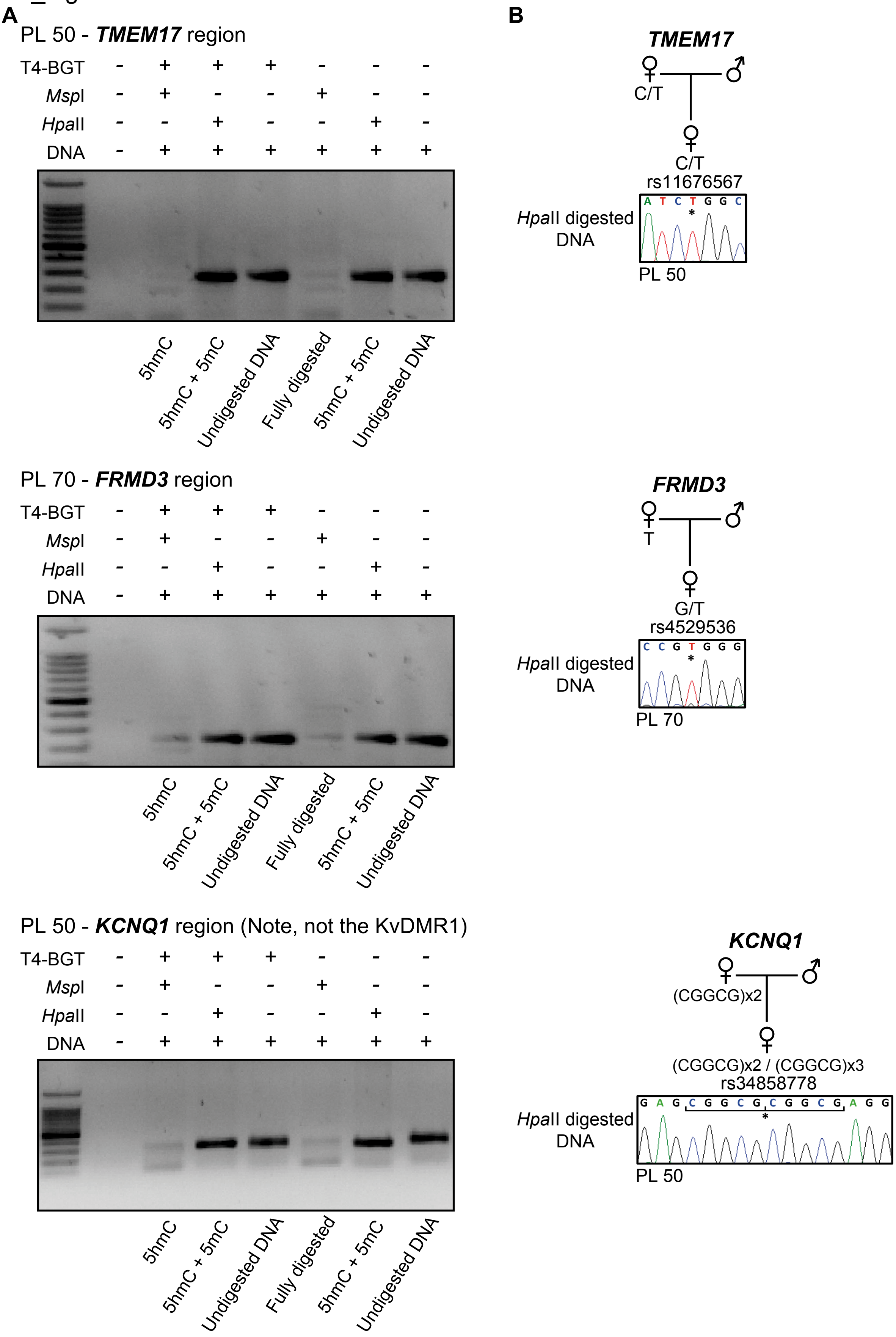
A
PL 50 - TMEM17 region
T4-BGT - + + + - - -
MspI - + - - + - -
HpaII - - + - - + -
DNA - + + + + + +
5hmC
5hmC + 5mC
Undigested DNA
Fully digested
5hmC + 5mC
Undigested DNA
PL 70 - FRMD3 region
T4-BGT - + + + - - -
MspI - + - - + - -
HpaII - - + - - + -
DNA - + + + + + +
5hmC
5hmC + 5mC
Undigested DNA
Fully digested
5hmC + 5mC
Undigested DNA
PL 50 - KCNQ1 region (Note, not the KvDMR1)
T4-BGT - + + + - - -
MspI - + - - + - -
HpaII - - + - - + -
DNA - + + + + + +
5hmC
5hmC + 5mC
Undigested DNA
Fully digested
5hmC + 5mC
Undigested DNA
B
TMEM17
C/T
C/T
rs11676567
A T C T G G C
*
HpaII digested DNA
PL 50
FRMD3
T
G/T
rs4529536
C C G T G G G
*
HpaII digested DNA
PL 70
KCNQ1
(CGGCG)x2
(CGGCG)x2 / (CGGCG)x3
rs34858778
G A G C G G C G C G G C G A G G
*
HpaII digested DNA
PL 50
