# Supplementary material for: Human Oocyte-Derived Methylation Differences Persist in the Placenta Revealing Widespread Transient Imprinting
Source: PLoS Genet. 2016 Nov 11;12(11):e1006427. doi: 10.1371/journal.pgen.1006427 (PMC5106035; doi:10.1371/journal.pgen.1006427)
Supplement: S3 Table — (DOCX) [file pgen.1006427.s012.docx]

**S3_Table**

| **Gene** | **Number of heterozygous samples assessed by methylation-sensitive genotyping PCR** |
| --- | --- |
| *FRMD3* | 2 maternal, 2 uninformative monoallelic and 3 biallelically methylated placenta samples |
| *KCNQ1* | 8 maternal and 5 uninformative monoallelically methylated placenta samples |
| *CACNA1E* | 1 maternally methylated placenta sample |
| *TMEM247* | 2 maternally methylated placenta samples |
| ncRNA (close to *TET3)* | 5 maternal, 5 uninformative monoallelic and 3 biallelically methylated placenta samples |
| *SPHKAP* | 4 maternal, 1 uninformative monoallelic and 5 biallelically methylated placenta samples |
| *ZNF385D* | 2 maternal and 2 uninformative monoallelically methylated placenta samples |
| *C3ORF62* | 2 maternal, 1 uninformative monoallelic and 1 biallelically methylated placenta samples |
| *EFCC1* | 3 uninformative monoallelically methylated placenta samples |
| *FGF12* | 2 maternal, 1 uninformative monoallelic and 3 biallelically methylated placenta samples |
| *PDE6B* | 3 maternal and 1 uninformative monoallelically methylated placenta samples |
| *SH3BP2* | 2 uninformative monoallelically methylated placenta samples |
| *STX18-AS1* | 3 maternal and 1 uninformative monoallelically methylated placenta samples |
| *GPR78* | 1 maternal, 2 uninformative monoallelic and 1 biallelically methylated placenta samples |
| *GRID2* (promoter) | 1 maternal and 1 uninformative monoallelically methylated placenta samples |
| *SFRP2* | 3 maternal and 4 uninformative monoallelically methylated placenta samples |
| *DLGAP2* | 1 maternal and 3 uninformative monoallelically methylated placenta samples |
| *R3HCC1* | 4 maternal, 1 uninformative monoallelic and 3 biallelically methylated placenta samples |
| *DENND3* | 5 maternal, 3 uninformative monoallelic and 4 biallelically methylated placenta samples |
| *OPCML* | 1 maternal and 1 uninformative monoallelically methylated placenta samples |
| *CACNA1C* | 2 maternal and 3 uninformative monoallelically methylated placenta samples |
| *FGF14* | 7 maternal and 2 uninformative monoallelically methylated placenta samples |
| *PAPLN-AS1* | 2 maternal and 3 uninformative monoallelically methylated placenta samples |
| *RYR3* | 1 maternal and 1 uninformative monoallelically methylated placenta samples |
| *BOD1L2* | 4 uninformative monoallelically methylated placenta samples |
| Chr 18 | 3 maternal and 4 uninformative monoallelically methylated placenta samples |
| *CACNA1A* | 4 maternal, 1 uninformative monoallelic and 2 biallelically methylated placenta samples |
| *ACTL10* | 2 maternal and 1 uninformative monoallelically methylated placenta samples |
| *TPTEP1* | 5 maternal and 3 uninformative monoallelically methylated placenta samples |
| *CACNA1I* | 2 maternal and 4 uninformative monoallelically methylated placenta samples |
| Chr. 1 | 16 biallelic placenta samples |
| *THSD7B* | 10 biallelic placenta samples |
| *DPP6* | 11 biallelically methylated placenta samples |
| *SLC2A2* | 10 biallelically methylated placenta samples |
| *RPS6KAL* | 11 biallelically methylated placenta samples |
| *OPRM1* | 7 biallelically methylated placenta samples |
| *RADIL* | 8 biallelically methylated placenta samples |
| *NTNG2* | 7 biallelically methylated placenta samples |
| Chr. 10 | 3 biallelically methylated placenta samples |
| *UNC79* | 12 biallelically methylated placenta samples |
| *OCA2* | 5 biallelically methylated placenta samples |
| *FHOD3* | 1 biallelically methylated placenta sample |
| *GP6* | 10 biallelically methylated placenta samples |

The number of heterozygous placenta samples used to determine allelic methylation of novel imprinted DMRs.
