# Supplementary material for: Human Oocyte-Derived Methylation Differences Persist in the Placenta Revealing Widespread Transient Imprinting
Source: PLoS Genet. 2016 Nov 11;12(11):e1006427. doi: 10.1371/journal.pgen.1006427 (PMC5106035; doi:10.1371/journal.pgen.1006427)
Supplement: S4 Table — (DOCX) [file pgen.1006427.s013.docx]

**S4_Table**

| **Gene** | **Number of heterozygous samples assessed by allelic RT-PCR** |
| --- | --- |
| *SVOPL* | 2 maternally expressed in placenta samples, 2 monoallelically expressed in brain samples and 2 monoallelically expressed in lymphocyte samples. |
| *AGO1* | 2 paternally expressed and 1 monoallelically expressed from the unmethylated allele in placenta samples. |
| *C3ORF62* | 1 paternally expressed, 1 monoallelically expressed from the unmethylated allele and 1 biallelically expressed in placenta samples. |
| *SH3BP2* | 4 paternally expressed, 2 monoallelically expressed from the unmethylated allele and 5 biallelically expressed in placenta samples. |
| *FAM149A* | 1 paternally expressed in placenta samples. |
| *MOCS1* | 1 paternally expressed, 1 monoallelically expressed from the unmethylated allele and 1 biallelically expressed in placenta samples. |
| *R3HCC1* | 3 paternally expressed, 1 monoallelically expressed from the unmethylated allele and 4 biallelically expressed in placenta samples. |
| *JMJD1C* | 1 paternally expressed and 2 monoallelically expressed from the unmethylated allele in placenta samples. |
| *PAK1* | 2 monoallelically expressed from the unmethylated allele and 2 biallelically expressed in placenta samples. |
| *PAPLN-AS1* | 1 paternally expressed, 3 monoallelically expressed from the unmethylated allele and 1 biallelically expressed in placenta samples. |
| ncRNA (close to *TET3)* | 2 paternally expressed and 1 monoallelically expressed from the unmethylated allele in placenta samples. |
| *TET3* | 4 biallelically expressed in placenta samples. |
| *ZHX3* | 4 paternally expressed, 1 monoallelically expressed from the unmethylated allele and 3 biallelically expressed in placenta samples. |
| *LAMA2* | 8 biallelically expressed in placenta samples. |
| ncRNA (close to *LAMA2* | 3 biallelically expressed in placenta samples. |
| *DENND3* | 6 biallelically expressed in placenta samples. |
| *RNF141* | 10 biallelically expressed in placenta samples. |
| *TBC1D30* | 3 biallelically expressed in placenta samples. |
| *FGF14* | 2 biallelically expressed in placenta samples. |
| *SIAH1* | 11 biallelics in placenta samples. |
| *ACTL10* | 3 biallelically expressed in placenta samples. |

The number of heterozygous placenta samples used to determine allelic RT-PCR.
